# Molecular Assortment of *Lens* Species with Different Adaptations to Drought Conditions Using SSR Markers

**DOI:** 10.1371/journal.pone.0147213

**Published:** 2016-01-25

**Authors:** Dharmendra Singh, Chandan Kumar Singh, Ram Sewak Singh Tomar, Jyoti Taunk, Ranjeet Singh, Sadhana Maurya, Ashish Kumar Chaturvedi, Madan Pal, Rajendra Singh, Sarawan Kumar Dubey

**Affiliations:** 1 Division of Genetics, Indian Agricultural Research Institute, New Delhi-110 012, India; 2 National Research Centre on Plant Biotechnology, Indian Agricultural Research Institute, New Delhi-110 012, India; 3 Division of Plant Physiology, Indian Agricultural Research Institute, New Delhi-110 012, India; 4 Division of Soil Science and Agricultural Chemistry, Indian Agricultural Research Institute, New Delhi-110 012, India; 5 Regional centre, Central Soil and Water Conservation Research and Training Institute, Agra-24 8195, India; NBPGR, INDIA

## Abstract

The success of drought tolerance breeding programs can be enhanced through molecular assortment of germplasm. This study was designed to characterize molecular diversity within and between *Lens* species with different adaptations to drought stress conditions using SSR markers. Drought stress was applied at seedling stage to study the effects on morpho-physiological traits under controlled condition, where tolerant cultivars and wilds showed 12.8–27.6% and 9.5–23.2% reduction in seed yield per plant respectively. When juxtaposed to field conditions, the tolerant cultivars (PDL-1 and PDL-2) and wild (ILWL-314 and ILWL-436) accessions showed 10.5–26.5% and 7.5%–15.6% reduction in seed yield per plant, respectively under rain-fed conditions. The reductions in seed yield in the two tolerant cultivars and wilds under severe drought condition were 48–49% and 30.5–45.3% respectively. A set of 258 alleles were identified among 278 genotypes using 35 SSR markers. Genetic diversity and polymorphism information contents varied between 0.321–0.854 and 0.299–0.836, with mean value of 0.682 and 0.643, respectively. All the genotypes were clustered into 11 groups based on SSR markers. Tolerant genotypes were grouped in cluster 6 while sensitive ones were mainly grouped into cluster 7. Wild accessions were separated from cultivars on the basis of both population structure and cluster analysis. Cluster analysis has further grouped the wild accessions on the basis of species and sub-species into 5 clusters. Physiological and morphological characters under drought stress were significantly (*P* = 0.05) different among microsatellite clusters. These findings suggest that drought adaptation is variable among wild and cultivated genotypes. Also, genotypes from contrasting clusters can be selected for hybridization which could help in evolution of better segregants for improving drought tolerance in lentil.

## Introduction

Lentil (*Lens culinaris* Medik.), is an important cool season grain legume which is grown worldwide in semi-arid regions where yield loss due to drought stress is very high [1]. Drought which is a major abiotic stress and a serious agronomic problem hinders plant growth, development and ultimately affects its productivity. Although lentil is a hardy crop and requires less water for its growth, yet under drought stress conditions, a considerable decrease in productivity can range between 6.0–54.0% [2]. Lentil seeds absorb water equal to their weight in less than 36 hours and germinate soon after, but germination is reduced if dehydration occurs [3]. This makes crop sensitive to early season drought, particularly when planted shallow [3]. Unfavourable soil moisture at sowing time of lentil results in irregular seed emergence, which in turn affects the establishment of stand with negative effects on plant yield [4]. Therefore, phenotyping of well adapted genotypes to manage future drought stress challenges in lentil is an utmost requirement.

The degree of drought stress depends on its impact on physiological and biochemical processes which are reflection of changes at molecular levels and these influences the ability of plant to adapt to drought stress. Studies involving plant responses to drought stress have identified various morpho-physiological indicators for recording drought tolerance in plants. Osmotic adjustment is considered as an adaptation to water stress, by which an increase in the solute content of cells can lead to maintenance of turgor and turgor-related processes at low water potentials [5]. However, there are conflicting reports on the role of osmotic adjustment in maintenance of turgor and its association with seed yield [6, 7]. Several other parameters such as relative water content, water use efficiency, seedling vigour, stomatal conductance and chlorophyll content [8–15] have also been used to determine drought tolerance in plants. Singh *et al*. demonstrated that seedling survivability is an important parameter for assessing drought tolerance in crop plants [16].

Wild gene pool possesses excellent genetic potential that may eventually be exploited in cultivated types and thereby they are always on agenda as possible donors for drought resistance [17]. There is a need to evaluate genetic relatedness of wild genotypes with respect to cultivars as well as to characterize their genetic profile in relation to drought stress. Some wild accessions have good source of drought tolerance (Singh *et al*., Unpublished), and will be of great benefit in developing drought tolerant cultivars. All the wild *Lens* species are not crossable to the cultigens but the crossability between *L*. *culinaris orientalis* and *L*. *odomensis* with cultivated lentil is well studied, which offers a tremendous scope for improvement through interspecific hybridization and pre-breeding [18, 19]. However, there is difficulty in obtaining hybrids of the cultigens with *L*. *nigricans* and *L*. *ervoides*. Some viable hybrids have been reported between cultivated species and *L*. *ervoides*, *L*. *odomensis* and *L*. *nigricans* with the use of GA_3_ [20].

The success of breeding depends on genetic variability among the parental lines [21], as lack of this may limit breeding progress and the gain from selection [22]. The employment of genetic variation identified by DNA based molecular markers in plant breeding programs may be useful in addressing abiotic stresses during crop production. Some DNA based molecular markers such as restriction fragment length polymorphism (RFLP), amplified fragment length polymorphism (AFLP), inter simple sequence repeat (ISSR), random amplified polymorphic DNA (RAPD) and sequence-tagged microsatellite have been used to study genetic diversity in lentil [23–25]. However, there are limited studies involving use of microsatellite or simple sequence repeat (SSR) markers in lentil [26–31], that have several desirable features like robustness, high level of polymorphism, high reproducibility, co-dominance, giving them an advantage over other DNA based markers like RAPD and RFLP for applications in population genetics, genetic diversity studies, and DNA fingerprinting. Further, few studies using SSR have been conducted in lentil with different adaptation to drought stress conditions in cultivars, breeding lines, landraces and wild accessions [31]. Therefore, the objectives of this study were to characterize global collections of lentil accessions with different adaptations to drought conditions for their genetic variation present in wild and cultivated species using simple sequence repeat markers and to analyse drought tolerance among molecularly characterized *Lens* species based on morpho-physiological approaches.

## Materials and Methods

### Plant materials

A set of 278 genotypes including cultivars, advanced breeding lines, landraces and wild accessions were used for evaluation of drought tolerance in the controlled environments (Table 1). These genotypes represents diverse germplasm originating from ICARDA (128), India (61), Syria (25), Turkey (26), Israel (4), Italy, Spain, Jordan (3), Argentina, Slovenia, Lebanon, France, Bangladesh (2), Ethiopia, Croatia, Mexico, Nepal, Pakistan, Tajikistan, USA, Uzbekistan, Palestine and Azerbaijan (1). Origin of five genotypes was not known.

**Table 1:** Genotypes with different origins and sensitivity to drought stress.

S.N.	Genotype	Origin	Type	DR	S.N.	Genotype	Origin	Type	DR
1	ILL-10857	ICARDA	GC	MT	140	ILL-4404	Pakistan	GC	MT
2	121–12	India	GC	MT	141	ILL-4605	Argentina	Cult.	MT
3	1220–11	India	BL	MS	142	ILL-560	Turkey	GC	MT
4	210–11	India	BL	MS	143	ILL-5722	ICARDA	GC	MT
5	330–12	India	GC	MS	144	ILL-5883	Jordan	GC	MT
6	DPL-62	India	Cult.	MS	145	ILL-590	Turkey	GC	MT
7	E-153	India	GC	MS	146	ILL-6002	ICARDA	GC	T
8	FLIP-96-51	ICARDA	GC	T	147	ILL-76037	ICARDA	GC	T
9	IG-109039	ICARDA	GC	T	148	ILL-7978	ICARDA	GC	MT
10	IG-111991	ICARDA	LR	MS	149	ILL-7979	ICARDA	GC	MT
11	IG-111996	ICARDA	LR	MS	150	ILL-7982	ICARDA	GC	MT
12	IG-112078	ICARDA	LR	MS	151	ILL-8006	Bangladesh	GC	MT
13	IG-11210	ICARDA	LR	MS	152	ILL-8108	Argentina	GC	MT
14	IG-112128	ICARDA	LR	MS	153	ILL-8329	ICARDA	GC	MS
15	IG-112131	ICARDA	LR	MS	154	ILL-91887	ICARDA	GC	MT
16	IG-112137	ICARDA	LR	MS	155	ILL-9841	ICARDA	GC	T
17	IG-116551	ICARDA	LR	MS	156	ILL-9900	ICARDA	GC	MT
18	IG-129185	ICARDA	LR	MS	157	ILL-9916	ICARDA	GC	T
19	IG-129214	ICARDA	LR	MS	158	ILL-9941	ICARDA	GC	MT
20	IG-129287	ICARDA	LR	MS	159	ILL-9960	ICARDA	GC	MT
21	IG-129291	ICARDA	LR	MS	160	ILWL-06	Turkey	Wild	MS
22	IG-129293	ICARDA	LR	MS	161	ILWL-09	Syria	Wild	MS
23	IG-129302	ICARDA	LR	MS	162	ILWL-10	-	Wild	MT
24	IG-129304	ICARDA	LR	MS	163	ILWL-100	Turkey	Wild	MS
25	IG-129309	ICARDA	LR	MS	164	ILWL-104	Turkey	Wild	MS
26	IG-129313	ICARDA	LR	MS	165	ILWL-125	Syria	Wild	MS
27	IG-129315	ICARDA	LR	S	166	ILWL-128	Syria	Wild	MT
28	IG-129317	ICARDA	LR	MS	167	ILWL-13	ITA	Wild	MS
29	IG-129319	ICARDA	LR	MS	168	ILWL-133	Syria	Wild	MS
30	IG-129372	ICARDA	LR	MS	169	ILWL-137	Syria	Wild	MT
31	IG-129560	ICARDA	LR	MS	170	ILWL-142	Syria	Wild	MS
32	IG-12970	ICARDA	LR	MS	171	ILWL-15	France	Wild	MS
33	IG-130033	ICARDA	LR	MS	172	ILWL-165	Syria	Wild	MS
34	IG-130219	ICARDA	LR	MS	173	ILWL-184	Syria	Wild	MT
35	IG-130272	ICARDA	LR	MS	174	ILWL-185	Syria	Wild	MT
36	IG-134342	ICARDA	LR	MS	175	ILWL-192	Syria	Wild	MT
37	IG-134347	ICARDA	LR	MS	176	ILWL-20	Palestine	Wild	MS
38	IG-134356	ICARDA	LR	MS	177	ILWL-203	Turkey	Wild	MS
39	IG-135424	-	Wild	MS	178	ILWL-221	Turkey	Wild	MS
40	IG-135428	-	Wild	MS	179	ILWL-227	Syria	Wild	MT
41	IG-136607	ICARDA	LR	MS	180	ILWL-23	Italy	Wild	MS
42	IG-136608	-	Wild	MS	181	ILWL-237	Syria	Wild	MS
43	IG-136612	Turkey	Wild	MS	182	ILWL-238	Syria	Wild	MS
44	IG-136614	Italy	Wild	MT	183	ILWL-253	Syria	Wild	MT
45	IG-136618	Croatia	Wild	MS	184	ILWL-269	Turkey	Wild	MS
46	IG-136620	Slovenia	Wild	MT	185	ILWL-29	Spain	Wild	MS
47	IG-136626	Israel	Wild	MT	186	ILWL-292	Turkey	Wild	MT
48	IG-136637	France	Wild	MS	187	ILWL-3	Turkey	Wild	MT
49	IG-136652	Israel	Wild	MS	188	ILWL-314	Turkey	Wild	T
50	IG-136653	Israel	Wild	MS	189	ILWL-320	Turkey	Wild	MT
51	IG-136673	Turkey	Wild	MS	190	ILWL-321	Turkey	Wild	MT
52	IG-136788	Syria	Wild	MS	191	ILWL-334	Jordan	Wild	MS
53	IG-140910	Azerbaijan	Wild	MS	192	ILWL-340	Jordan	Wild	MS
54	IG-149	ICARDA	LR	MS	193	ILWL-35	Turkey	Wild	MS
55	IG-49	ICARDA	LR	MS	194	ILWL-350	Syria	Wild	MS
56	IG-5320	ICARDA	LR	MS	195	ILWL-357	Syria	Wild	MT
57	IG-69540	ICARDA	LR	MT	196	ILWL-361	Syria	Wild	MS
58	IG-69549	ICARDA	LR	MS	197	ILWL-362	Syria	Wild	MT
59	IG-70174	ICARDA	LR	MS	198	ILWL-366	Syria	Wild	MT
60	IG-70230	ICARDA	LR	MS	199	ILWL-370	Syria	Wild	MT
61	IG-71352	ICARDA	LR	MS	200	ILWL-377	Tajiskistan	Wild	MT
62	IG-71630	ICARDA	LR	MS	201	ILWL-398(A)	Lebanon	Wild	MS
63	IG-71646	ICARDA	LR	MS	202	ILWL-401	Lebanon	Wild	MT
64	IG-71685	ICARDA	LR	MS	203	ILWL-415	Syria	Wild	MS
65	IG-71710	ICARDA	LR	MS	204	ILWL-418	Syria	Wild	MS
66	IG-73717	ICARDA	LR	MS	205	ILWL-428	Spain	Wild	MS
67	IG-73798	ICARDA	LR	MS	206	ILWL-430	Spain	Wild	MS
68	IG-73802	ICARDA	LR	MS	207	ILWL-436	Turkey	Wild	T
69	IG-73816	ICARDA	LR	MS	208	ILWL-437	Turkey	Wild	T
70	IG-73945	ICARDA	LR	MS	209	ILWL-438	Turkey	Wild	MT
71	IG-75920	ICARDA	LR	MS	210	ILWL-44	Slovenia	Wild	MT
72	IG-9	ICARDA	LR	MS	211	ILWL-447	Turkey	Wild	MS
73	IG-936	ICARDA	LR	MS	212	ILWL-462	Turkey	Wild	T
74	ILL-358	Mexico	GC	MT	213	ILWL-464	Syria	Wild	MT
75	ILL-7349	Nepal	GC	MT	214	ILWL-468	Syria	Wild	MT
76	ILL-9896	USA	GC	T	215	ILWL-472	-	Wild	MS
77	ILL-10030	ICARDA	GC	MT	216	ILWL-55(2)	Israel	Wild	T
78	ILL-10031	ICARDA	GC	MT	217	ILWL-58	Turkey	Wild	MT
79	ILL-10032	ICARDA	GC	MT	218	ILWL-60	Turkey	Wild	MT
80	ILL-10034	ICARDA	GC	MT	219	ILWL-69	Uzbekistan	Wild	MS
81	ILL-10040	ICARDA	GC	MT	220	ILWL-83	Turkey	Wild	MS
82	ILL-10041	ICARDA	GC	MT	221	ILWL-95	Turkey	Wild	MS
83	ILL-10043	ICARDA	GC	MT	222	IPL-406	India	Cult.	MS
84	ILL-10056	ICARDA	GC	MT	223	JL-3	India	Cult.	S
85	ILL-10062	ICARDA	GC	MT	224	L-404	India	BL	MS
86	ILL-10063	ICARDA	GC	MT	225	L-4076	India	Cult.	MS
87	ILL-10074	ICARDA	GC	MT	226	L-4078	India	BL	MS
88	ILL-10075	ICARDA	GC	MT	227	L-4147	India	Cult.	MS
89	ILL-10082	ICARDA	GC	MT	228	L-4578	India	BL	MS
90	ILL-10133	ICARDA	GC	MT	229	L-4590	India	Cult.	MS
91	ILL-10234	ICARDA	GC	MT	230	L-4594	India	Cult.	MS
92	ILL-10266	ICARDA	GC	MT	231	L-4603	India	BL	MS
93	ILL-10270	ICARDA	GC	MT	232	L-4605	India	BL	MS
94	ILL-1046	ICARDA	GC	MT	233	L-4618	India	BL	MS
95	ILL-10756	ICARDA	GC	MT	234	L-4619	India	BL	MS
96	ILL-10794	ICARDA	GC	MT	235	L-4620	India	BL	MS
97	ILL-10795	ICARDA	GC	MT	236	L-4650	India	BL	MS
98	ILL-10804	ICARDA	GC	MT	237	L-4701	India	BL	MS
99	ILL-10805	ICARDA	GC	MT	238	L-5253	India	BL	MS
100	ILL-10806	ICARDA	GC	MT	239	L-7752	India	BL	MS
101	ILL-10807	ICARDA	GC	MT	240	L-7818	India	BL	MS
102	ILL-10809	ICARDA	GC	MT	241	L7905	India	BL	MS
103	ILL-10810	ICARDA	GC	MT	242	L-7920	India	BL	MS
104	ILL-10811	ICARDA	GC	MT	243	LC-270-804	India	BL	MS
105	ILL-10812	ICARDA	GC	MT	244	LC-282-1077	India	BL	MS
106	ILL-10817	ICARDA	GC	MT	245	LC-282-1110	India	BL	MS
107	ILL-10818	ICARDA	GC	MT	246	LC-282-1444	India	BL	MS
108	ILL-10819	ICARDA	GC	MT	247	LC-282-896	India	BL	MS
109	ILL-10820	ICARDA	GC	MT	248	LC-284-116	India	BL	MS
110	ILL-10823	ICARDA	GC	MT	249	LC-284-1209	India	BL	MS
111	ILL-10826	ICARDA	GC	MT	250	LC-285-1344	India	BL	MS
112	ILL-10827	ICARDA	GC	MT	251	LC-289-1444	India	BL	MS
113	ILL-10831	ICARDA	GC	MT	252	LC-289-1447	India	BL	MS
114	ILL-10834	ICARDA	GC	MT	253	LC-292-1485	India	BL	MS
115	ILL-10835	ICARDA	GC	MT	254	LC-292-1544	India	BL	MS
116	ILL-10836	ICARDA	GC	MT	255	LC-292-997	India	BL	MS
117	ILL-10837	Turkey	GC	MT	256	LC-300-11	India	BL	MS
118	ILL-10848	Bangladesh	GC	MT	257	LC-300-12	India	BL	MS
119	ILL-10893	ICARDA	GC	T	258	LC-300-13	India	BL	MS
120	ILL-10894	ICARDA	GC	MT	259	LC-300-15	India	BL	MS
121	ILL-10897	ICARDA	GC	MT	260	LC-300-16	India	BL	MS
122	ILL-10913	ICARDA	GC	MT	261	LC-300-2	India	BL	MS
123	ILL-10915	ICARDA	GC	MT	262	LC-300-3	India	BL	MS
124	ILL-10917	ICARDA	GC	MT	263	LC-300-4	India	BL	MS
125	ILL-10921	ICARDA	GC	MT	264	LC-300-6	India	BL	MS
126	ILL-10922	ICARDA	GC	MT	265	LC-300-7	India	BL	MS
127	ILL-10951	ICARDA	GC	MT	266	LC-300-8	India	BL	MS
128	ILL-10953	ICARDA	GC	MT	267	LC-300-9	India	BL	MS
129	ILL-10960	ICARDA	GC	MT	268	LC-74-1-51	India	BL	MS
130	ILL-10961	ICARDA	GC	MT	269	PDL-1	ICARDA	BL	T
131	ILL-10963	ICARDA	GC	MT	270	PDL-2	ICARDA	BL	T
132	ILL-10964	ICARDA	GC	MT	271	PKVL-1	India	Cult.	MS
133	ILL-10965	ICARDA	GC	MS	272	PL-1	India	Cult.	MS
134	ILL-10967	ICARDA	GC	MT	273	PL-4	India	Cult.	MS
135	ILL-10969	ICARDA	GC	MT	274	PL-406	India	Cult.	MS
136	ILL-10970	ICARDA	GC	MT	275	PL-5	India	Cult.	MS
137	ILL-10972	ICARDA	GC	MT	276	Sehore-74-3	India	Cult.	MS
138	ILL-1970	Ethiopia	GC	MT	277	VL-507	India	Cult.	S
139	ILL-3829	ICARDA	GC	MT	278	WBL-77	India	Cult.	MS

DR, Drought reaction; GC, Germplasm collection; BL, Breeding line; LR, Landrace; Cult., Cultivar, T, Tolerant; MT, Moderately tolerant; MS, Moderately sensitive; S, Sensitive.

### Experimental details

#### Evaluation of genotypes in hydroponic assay

Hydroponic experiment was conducted under controlled environment, at National Phytotron Facility, Indian Agricultural Research Institute, New Delhi, India in a completely randomized design with three replications. Air temperature in the National Phytotron Facility was 22/18°C (±2°C) day/night; photoperiod was 10/14 h light/dark; and the relative humidity was approximately 45%. Drought tolerance was assayed following the protocol of Singh *et al*. [16]. Seeds were disinfected with 1% sodium hypochlorite for 2–3 min, rinsed thoroughly with distilled water and then germinated on filter paper. After 1 week, twelve seedlings per replicate were transferred to hydroponic medium. The composition of hydroponic medium was as per nutrient composition of Simon *et al*. [32]. After 1 week of transferring the seedlings in hydroponic medium, drought stress was imposed by removing them from the nutrient solution such that their roots remained exposed to air for a period of 5 h daily for 6 consecutive days. Control plants were kept in the nutrient solution for the entire period (6 days) of development without interruption. The pH of the nutrient solution was adjusted to 6.5 using 1 M HCl or 1 M KOH. The solution was regularly aerated by bubbling air with an aquarium air pump and was replaced after every 4 days. After 6 days of treatment, drought tolerance indexes were determined for the seedling survivability and drought score. Seedling survivability was calculated as follows: Seedling survivability % = ratio of seedlings which survived the drought stress to the total number of seedlings used in the experiment x 100. Drought tolerance was estimated by the wilting score (WS) as the degree of wilting severity using the following 0–4 score scale as described by Singh *et al*. and Idrissi *et al*. [16, 33]: 0 = healthy plants with no visible symptoms of drought stress; 1 = green plants with slight wilting; 2 = leaves turning yellowish green with moderate wilting; 3 = leaves yellow—brown with severe wilting and 4 = completely dried leaves and/or stems. The reduction of root length and shoot length, fresh and dry weights was also recorded after 6 days of air exposure at the seedling stage.

The non-survived (sensitive, where stems were green but leaves were all dead) plants showed recovery (new leaves started emerging) after 15 days in nutrient solution. The recovered (tolerant) and non recovered (sensitive) plants were transferred to field at normal conditions till their maturity period. For comparison, the control plants without stress were also transferred to field for seed yield per plant. The reduction % was calculated by the following formula: (seed yield of stressed plants)-(seed yield of control plants)/seed yield of control plants x 100. The relative water content and chlorophyll contents were determined at seedling stage. The methods of relative water content and chlorophyll contents are presented below:

Relative water content was determined following the method described by Barrs and Weatherley [34]. Fresh weight of leaf samples (0.5 g) were taken in three replicates and kept in petriplates filled with distilled water for 4 hours. Thereafter, the samples were weighed (for turgid weight), oven dried and dry weight was determined. The percent RWC was calculated from following formula: RWC% = (Fresh weight—Dry weight)/ (Turgid weight—Dry weight) x 100.

Chlorophyll contents were measured by non-maceration method of Hiscox and Israelstam [35]. Fresh leaf samples (0.1 g) were added in test tubes containing 10 ml of Dimethyl sulfoxide (DMSO). To avoid exposure from light, these test tubes were covered with aluminium foil and kept in oven at 65°C for 4 hours. Subsequently the tubes were shaken to mix the pigments to distribute uniformly and the absorbance was measured at 645 and 663 nm in UV visible spectrophotometer (Model 5000, Perkin-Elmer, Shelton, CT-USA). The amount of chlorophyll a, chlorophyll b and total chlorophyll contents were calculated using the formulas provided by Mac Kinney, Arnon and Richardson *et al*. [36–38].

#### Growth conditions for field experiments

The field experiments were undertaken at IARI, New Delhi and CSWCRTI, Agra. The experiments were conducted in completely randomized design with three replications. The soil textures were sandy and loamy types at IARI, New Delhi and CSWCRTI, Agra at 0–15 cm soil depth, respectively. Organic carbon %, pH and electrical conductivity were 4.9 g/kg, 7.9, 0.35 dsm^-1^ and 3.8g/kg, 8.46, 0.22 dsm^-1^ at 0–15 cm soil depth at IARI and CSWCRTI, respectively. Particle size distribution (%) was 56, 24, 20 and 14, 21, 65 at 0–15 cm soil depth at IARI and CSWCRTI for sand, silt and clay, respectively.

Soil moisture content was determined by gravimetric method as: Moisture content (%) = Weight of wet soil-weight of dry soil/weight of dry soil x 100. During rain-fed conditions, soil moisture content was 22.3% and 24.7% (0–15 cm soil depth) at sowing time in IARI and CSWCRTI, respectively. The soil moisture content at the vegetative, reproductive and maturity stages was 14.1% and 19.2%; 13.2% and 16.1%; 10.8% and 12.4% at 0–15 and 15–30 cm soil depth, respectively at IARI under rainfed conditions. It was 15.3% and 20.4%, 14.6% and 17.2% and 11.1% and 14.2% at 0–15 and 15–30 cm soil depth at vegetative, reproductive and maturity stages, respectively, at CSWCRTI, Agra under rainfed conditions. During severe drought condition, it was 23.9% (0–15 cm) at sowing time, 21.4% (0–15 cm soil depth) and 23.6% (15–30 cm soil depth) at vegetative stage, it was 16.1% at 0–15 cm soil depth and 20.2% at 15–30 cm soil depth at reproductive stage and 10.2% (0-15cm soil depth) and 14.9% (15-30cm soil depth) at maturity stage at IARI.

#### Evaluation of genotypes under severe stress conditions in field assay

The field experiment under severe stress condition was conducted during 2013–14 at Research Farm of Indian Agricultural Research Institute, New Delhi for confirmation of drought tolerance in cultivated and wild genotypes. Drought stress was imposed by covering plots with polythene tunnels, allowing the crop to grow solely on the stored soil moisture. For this, four polythene-covered tunnels were constructed. Each tunnel was used as block and each block was splited into two main plots (full irrigation or drought) where full irrigation was used as control. The experiment was conducted in completely randomized design with three replications.

#### Evaluation of genotypes in rain-fed condition in field assay

The experiments were carried out at the experimental fields of Central Soil and Water Conservation for Training and Research Institute, Agra, India during 2013–14 and 2014–15 and Indian Agricultural Research Institute, New Delhi, India during 2013–14 growing seasons under rain-fed conditions. In natural condition no irrigation water was provided from sowing till maturity and the crop solely depended on the rainfall. The average amount of the rainfall were 22.5 mm (2013–14) and 29.5 (2014–15) in Agra, and 1.1 mm in Delhi, respectively. Maximum and minimum temperature and relative humidity of each month is presented in S1 and S2 Tables. Field experiments were conducted in a randomized block design with three replications. Each experimental plot was comprised of 6 rows of 5 m length with inter and intra row spacing of 20 cm and 2.5 cm, respectively. The crop was raised without irrigation following the recommended cultural practices.

#### DNA extraction

Isolation of genomic DNA from the leaf samples of different genotypes (10 plants per individual) was performed by using conventional CTAB method described by Doyle and Doyle [39]. To check the quantity, it was compared with lambda uncut DNA on 1% agarose gel and quality was determined by using spectrophotometer. Standard working concentration of 50 ng/μl of DNA sample was used.

#### SSR marker analysis

Genetic diversity analysis was performed using a total of 495 SSR primers reported by Hamwieh *et al*., Kaur *et al*. and Jain *et al*. [40–42]. These markers were assayed for identification of polymorphism between drought tolerant (PDL-2) and drought sensitive (JL-3) genotypes (Singh *et al*., unpublished). Thirty five SSR primers exhibiting polymorphism between these two contrasting genotypes were used for further analysis of genetic diversity among 278 genotypes which included breeding lines, landraces, germplasm collection, cultivars and wild genotypes. The primers were synthesized by Microgen, South Korea and IDT, USA. Polymerase chain reaction was performed in 10μl reaction mixture comprising of 1 X PCR buffer, 0.1 U *Taq* DNA polymerase, 1 μl dNTP (1 mM), 0.5 μl of forward and reverse primers each (10 pM) (Microgen, South Korea and IDT, USA) and 50 ng/μl of genomic DNA in a thermocycler (Agilent Technologies, USA). The PCR protocol comprised of initial denaturation step of 94°C for 3 min followed by 40 cycles of 94°C for 1 min, annealing at 55°C for 30 sec, elongation at 72°C for 30 sec. with final extension at 72°C for 10 min. The amplified products were resolved on 3% ultra high resolution agarose gels and documented using Syngene Gel Documentation System.

#### Genetic diversity analysis

The genetic profile of 278 lentil genotypes was scored on the basis of difference in allele size using 35 SSR markers. To have a comparative view, the genetic profile of wild and cultivated genotypes was also performed separately. The major allele frequency, polymorphism information content (PIC) and genetic distance based clustering was performed with Unweighted Pair Group Method for Arithmetic average (UPGMA) tree using Power Marker v3.25 software [43] and the dendrogram was constructed using MEGA 4.0 software [44]. SSR data was again subjected to cluster analysis followed by bootstrap analysis with 1000 permutations for all the genotypes using Mega 4.0 software. The population structure for 278 lentil genotypes comprising both wilds and cultivars was inferred using Structure 2.3.4 software [45]. The structure outputs were visualized using Structure Harvester from which Evanno plots were constructed [46, 47]. An assumed admixed model with independent allele frequency and a uniform prior probability of the number of populations, *K* was used in structure. All the runs were conducted for *K* = 1 to 10 with 50,000 MCMC replicates after a burn-in of 50,000 replicates. For each value of *K*, 5 independent runs were done to generate an estimate of the true number of sub-populations [45]. The relation between genetic similarity identified by SSR markers and taxonomic distance measured by mean genetic distance and seedling survivability were analysed using Jaccard’s Similarity Index and average taxonomic distance was calculated by NTSYS-pc v2.1 software [48]. Duncan’s Multiple Range Test (DMRT) (*P* = 0.05) was used to evaluate differences among clusters for significance by using SPSS ver. 19.0 software.

## Results

### Phenotyping for drought tolerance

#### Hydroponic assay

**Fig 1:**
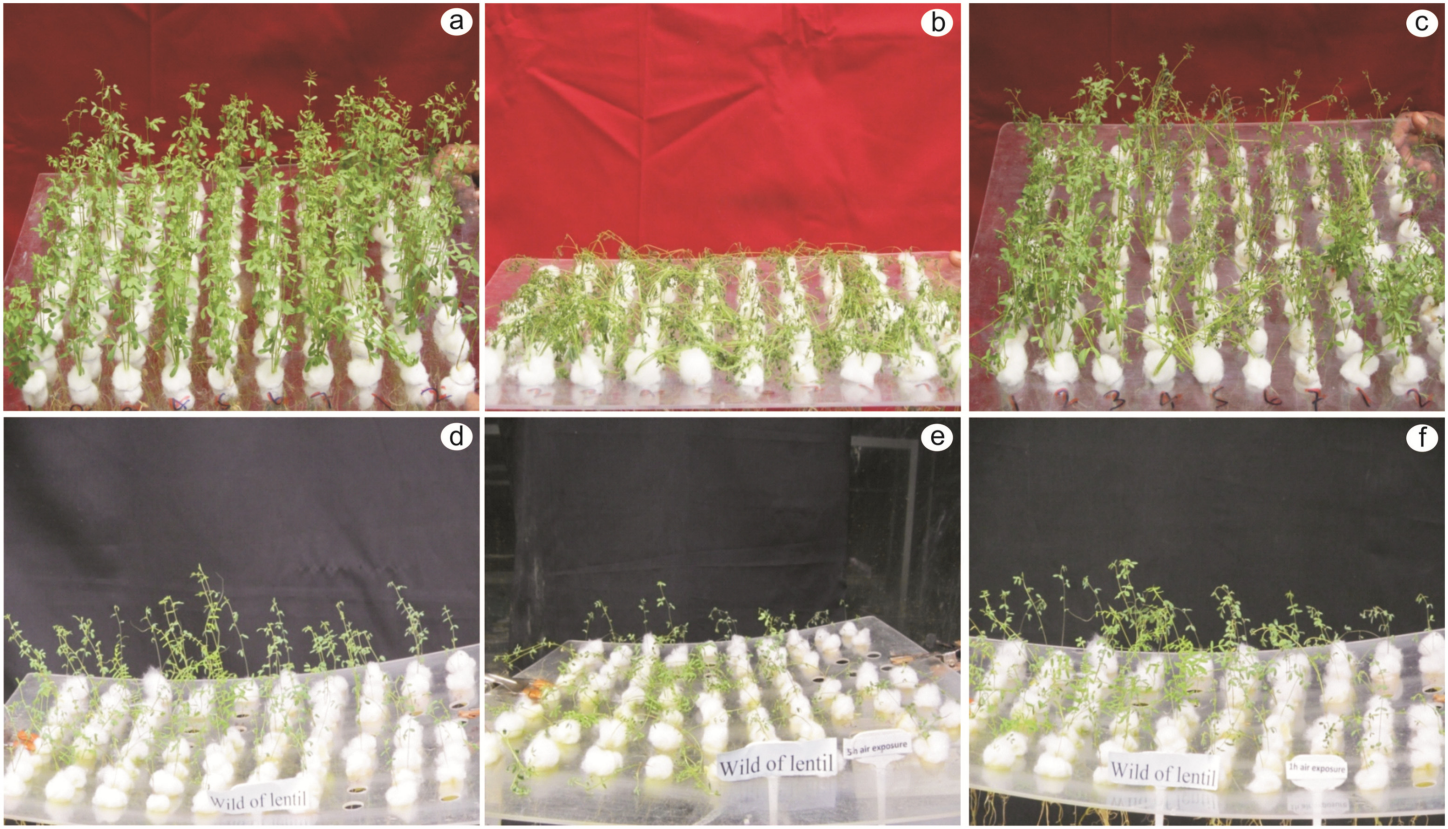
Evaluation of drought stress tolerance in cultivated and wild genotypes of lentil. Fifteen and 25 d old plants of cultivated and wild genotypes of lentil (a and d). Plant roots exposed to air for 5h (b and e). Recovery of genotypes in the nutrient solution (c and f).

**Fig 2:**
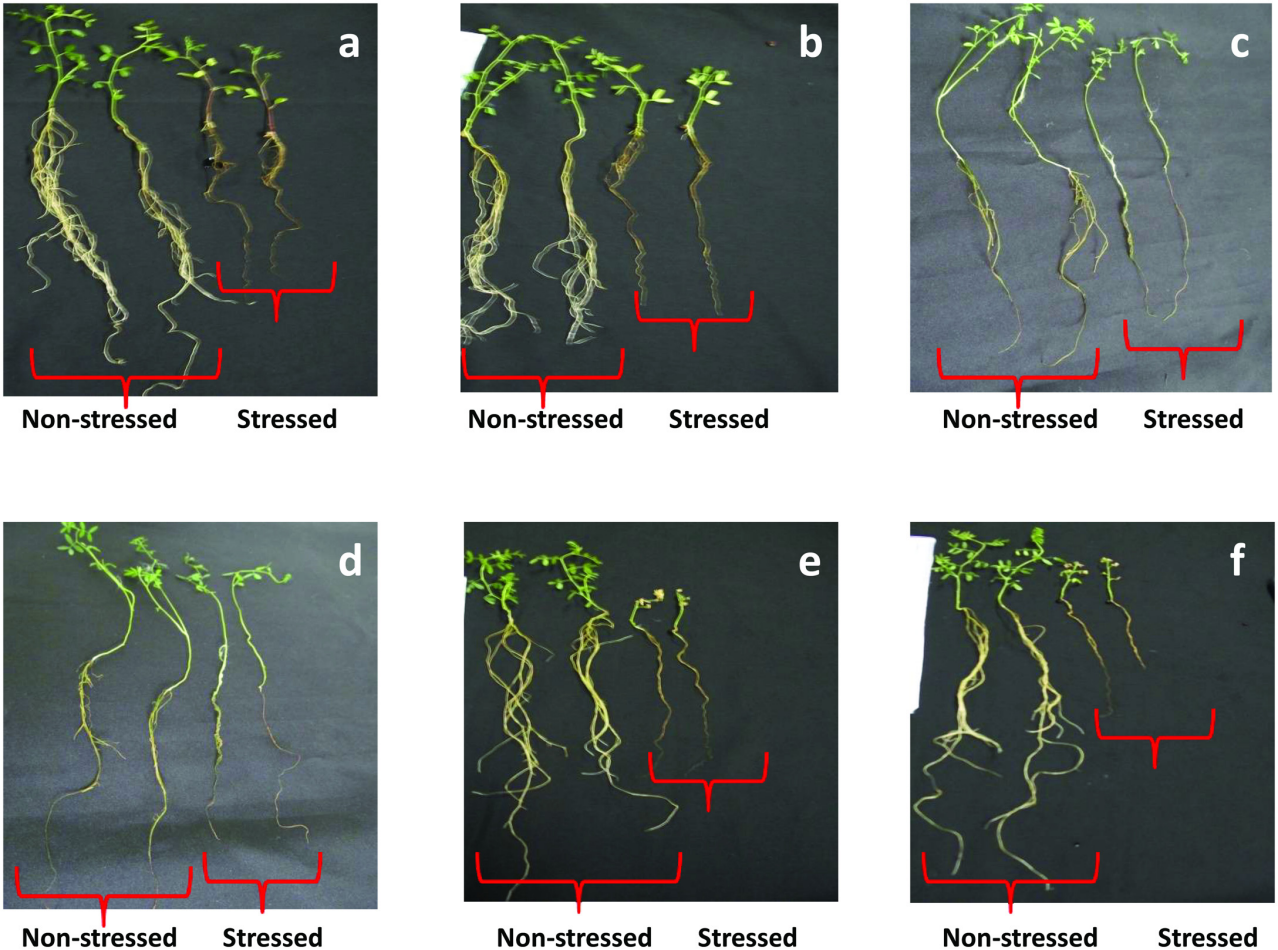
Recovery of tolerant and sensitive plants after 4 day air exposure. **Tolerant genotypes** [PDL-1 (a), PDL-2 (b), ILWL-334 (c), ILWL-436 (d)]; Sensitive genotypes [JL-3 (e), E-153 (f)].

Highly significant differences were found in the seedling survivability, drought score, reduction in root and shoot length, fresh and dry weights of roots and shoots and seed yield per plant. The effects of stress were first observed after 2 hours of air exposure when leaves were wilted in all the genotypes but no symptoms appeared in tolerant wild accessions (Figs 1 and 2). When plants were returned into the nutrient solution after 5 hours of air exposure and kept in nutrient solution for 12 hours, tolerant genotypes were fully recovered, moderately tolerant genotypes showed intermediate recovery while sensitive genotypes did not recovered at all. Differences among the genotypes became conspicuously visible and were progressively more pronounced with advancing days of roots exposure to air. Complete leaf death began on 6^th^ day in sensitive genotypes while tolerant wild accessions and tolerant cultivars did not show any mortality. On the basis of seedling survivability, drought score and growth parameters, the genotypes were assorted into four groups *viz*. tolerant, moderately tolerant, moderately sensitive and sensitive (Table 1). The genotypes which had drought score values in between 0–1 were classified as tolerant and those with 3–4 were classified as sensitive. Tolerant genotypes had seedling survivability of 71–100%, while in sensitive ones; it was 0–30% only. Genotypes with moderate drought score were further classified into moderately tolerant and moderately sensitive genotypes, on the basis of seedling survivability and reduction % in RWC. Moderately tolerant genotypes, had seedling survivability 51–70% and reduction in RWC in between 26–50%, while the respective values for moderately sensitive genotypes were 31–50% and 51–80%.

The lentil genotypes showed differential response to drought stress in terms of seedling growth reduction over the control. The tolerant genotypes showed 20.2–39.1% reduction in root length, 42.2–54.0% reduction in shoot length, 57–66.9% reduction in fresh root weight, 23.9–38.0% reduction in fresh shoot weight, 18.0–28.4% reduction in dry root weight and 17.6–33.2% reduction in dry shoot weight. Also, the tolerant lines showed 72.2–100% seedling survivability with a score of 0.0–1.0. However, sensitive genotypes showed 48.6–63.7% reduction in root length, 62.4–81.1% reduction in shoot length, 74.8–88.5% reduction in fresh root weight, 56.6–68.7% reduction in fresh shoot weight, 55.0–64.3% reduction in dry root weight, 72.0–81.6% reduction in dry shoot weight with a score 3.0–4.0 and seedling survivability of 0.0–36.2%. The survived tolerant genotypes exhibited 12.8–27.6% reduction in seed yield per plant when exposed to drought stress at seedling stage. However, sensitive genotypes showed 72.7–100% yield reduction in seed yield per plant while moderately tolerant and sensitive genotypes showed intermediate and low reduction in seed yield per plant respectively. In case of wild accessions, some of the tolerant genotypes showed 17.5–19.7% reduction in root length, 36.5–37.5% reduction in shoot length, 50.6–51.6% reduction in fresh root weight, 20.7–22.0% in fresh shoot weight, 15.8–18.8% in dry root weight and 16.3–17.6% in dry shoot weight. These tolerant lines showed 100% seedling survivability with a score of 0.0–0.2 and 9.5–23.2% yield reduction in seed yield per plant.

### SSR molecular marker analysis in *Lens* species

**Fig 3:**
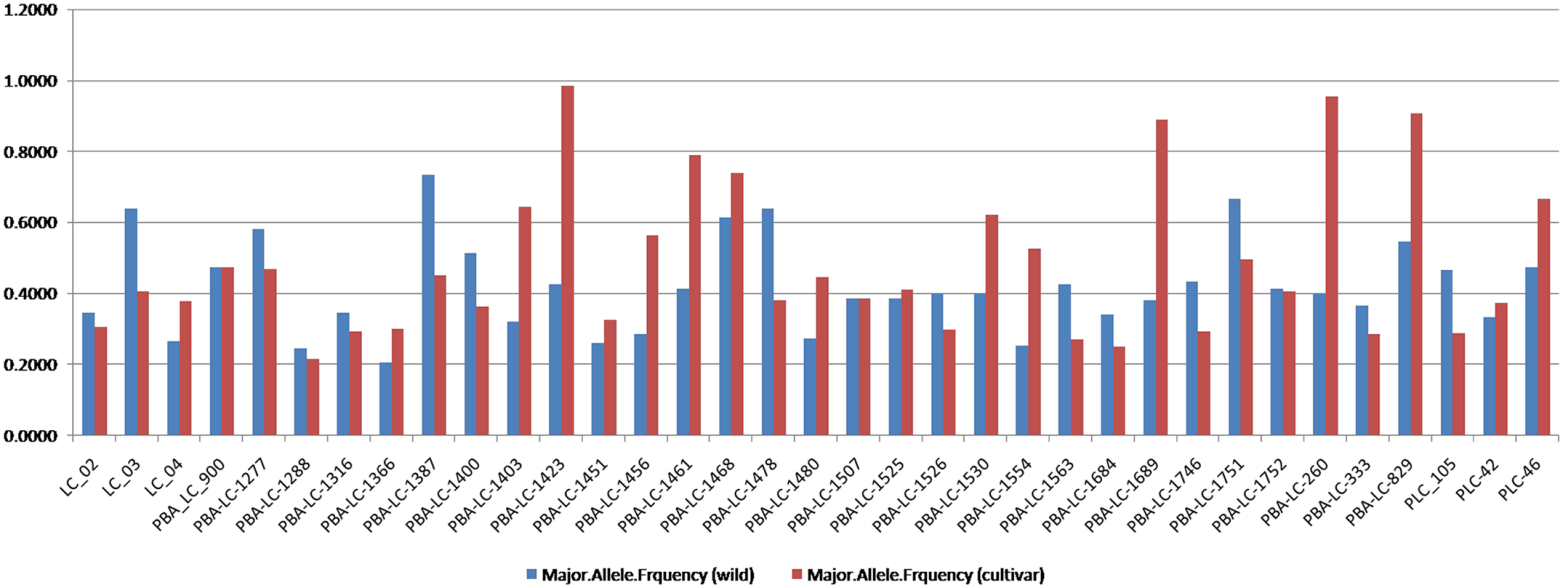
Major allele frequency for microsatellite loci (SSR) in wild and cultivated genotypes.

A set of 495 primers were pre-screened in drought tolerant (‘PDL-2’) and sensitive (‘JL-3’) genotypes, of which 35 SSR primers which exhibited polymorphism were selected for genetic diversity analysis among 278 genotypes (Table 2). All the 35 SSR primers generated polymorphic bands among the genotypes (Fig 3). A total of 258 alleles were identified with an average of 7.37 alleles per locus. The number of alleles per locus ranged from 4 (PBA_LC_1387, PBA_LC_1751 and PBA_LC_829) to 14 (LC_04). The gene diversity and PIC values varied between 0.321–0.854 and 0.299–0.836, with an average of 0.682 and 0.643, respectively. The primer which showed highest gene diversity and PIC values was PBA_LC_1288 while the lowest gene diversity and PIC values was observed for the primer PBA_LC_1423. Heterozygosity in all the genotypes ranged from 0 to 0.695 with a mean value of 0.102 and the highest heterozygosity was observed in PBA_LC_1400 (Table 2). The major allele frequency varied between 0.182 (PBA_LC_1288) to 0.809 (PBA_LC_1423) with a mean value of 0.429 (Fig 3).

**Table 2:** Allelic variation and PIC values for microsatellite loci (SSR) identified in 278 lentil genotypes.

Marker	Cultivars				Wild				All genotypes			
	AlleleNo.	Gene Diversity	H	PIC	Allele No.	Gene Diversity	H	PIC	Allele No.	Gene Diversity	H	PIC
LC_02	8.00	0.787	0.196	0.756	8.00	0.754	0.093	0.716	10.00	0.811	0.168	0.784
LC_03	10.00	0.677	0.338	0.622	10.00	0.569	0.013	0.551	12.00	0.778	0.251	0.746
LC_04	12.00	0.774	0.078	0.745	9.00	0.820	0.067	0.796	14.00	0.817	0.075	0.795
PBA_LC_1277	6.00	0.658	0.083	0.601	5.00	0.614	0.013	0.578	7.00	0.661	0.065	0.613
PBA_LC_1288	10.00	0.854	0.235	0.837	8.00	0.824	0.040	0.802	10.00	0.854	0.183	0.836
PBA_LC_1316	7.00	0.786	0.000	0.754	6.00	0.775	0.000	0.741	7.00	0.807	0.000	0.779
PBA_LC_1366	7.00	0.807	0.093	0.781	8.00	0.840	0.053	0.820	8.00	0.820	0.082	0.797
PBA_LC_1387	4.00	0.653	0.000	0.584	4.00	0.404	0.000	0.342	4.00	0.644	0.000	0.570
PBA_LC_1400	6.00	0.728	0.632	0.680	5.00	0.629	0.867	0.569	7.00	0.757	0.695	0.717
PBA_LC_1403	5.00	0.502	0.098	0.433	5.00	0.737	0.000	0.691	5.00	0.596	0.072	0.537
PBA_LC_1423	3.00	0.029	0.000	0.029	6.00	0.675	0.000	0.618	6.00	0.321	0.000	0.299
PBA_LC_1451	8.00	0.765	0.044	0.729	10.00	0.851	0.067	0.835	10.00	0.809	0.050	0.784
PBA_LC_1456	4.00	0.516	0.010	0.410	7.00	0.797	0.227	0.767	7.00	0.631	0.068	0.567
PBA_LC_1461	4.00	0.355	0.000	0.328	7.00	0.686	0.013	0.633	7.00	0.497	0.004	0.452
PBA_LC_1468	5.00	0.401	0.000	0.344	5.00	0.557	0.040	0.506	6.00	0.523	0.011	0.442
PBA_LC_1478	6.00	0.714	0.020	0.667	4.00	0.527	0.000	0.474	6.00	0.758	0.014	0.720
PBA_LC_1480	6.00	0.647	0.172	0.577	6.00	0.789	0.027	0.756	7.00	0.715	0.133	0.668
PBA_LC_1507	7.00	0.733	0.304	0.691	6.00	0.725	0.013	0.679	8.00	0.809	0.226	0.784
PBA_LC_1525	6.00	0.685	0.025	0.630	6.00	0.736	0.000	0.695	6.00	0.726	0.018	0.684
PBA_LC_1526	9.00	0.784	0.196	0.753	6.00	0.683	0.013	0.627	9.00	0.785	0.147	0.753
PBA_LC_1530	5.00	0.548	0.025	0.494	5.00	0.727	0.000	0.683	6.00	0.619	0.018	0.575
PBA_LC_1554	8.00	0.677	0.167	0.650	7.00	0.818	0.093	0.793	8.00	0.738	0.147	0.713
PBA_LC_1563	7.00	0.788	0.005	0.754	4.00	0.693	0.000	0.639	7.00	0.777	0.004	0.743
PBA_LC_1684	7.00	0.780	0.044	0.744	5.00	0.720	0.040	0.669	7.00	0.770	0.043	0.731
PBA_LC_1689	4.00	0.203	0.059	0.194	5.00	0.733	0.013	0.688	6.00	0.419	0.047	0.403
PBA_LC_1746	8.00	0.784	0.211	0.751	9.00	0.746	0.293	0.718	9.00	0.785	0.233	0.755
PBA_LC_1751	4.00	0.578	0.000	0.488	4.00	0.487	0.000	0.425	4.00	0.577	0.000	0.486
PBA_LC_1752	6.00	0.729	0.054	0.688	6.00	0.710	0.000	0.663	7.00	0.747	0.039	0.708
PBA_LC_260	4.00	0.090	0.059	0.089	6.00	0.722	0.040	0.679	8.00	0.343	0.054	0.330
PBA_LC_333	7.00	0.780	0.338	0.746	9.00	0.731	0.147	0.687	9.00	0.774	0.287	0.739
PBA_LC_829	3.00	0.171	0.000	0.161	4.00	0.620	0.000	0.568	4.00	0.431	0.000	0.385
PBA_LC_900	5.00	0.616	0.211	0.542	5.00	0.656	0.027	0.598	5.00	0.629	0.161	0.561
PLC_105	7.00	0.786	0.064	0.753	3.00	0.631	0.000	0.556	7.00	0.767	0.047	0.729
PLC_42	7.00	0.756	0.000	0.720	5.00	0.747	0.000	0.703	7.00	0.800	0.000	0.771
PLC_46	7.00	0.526	0.275	0.499	8.00	0.714	0.133	0.684	8.00	0.586	0.237	0.559
Mean	6.34	0.619	0.115	0.578	6.17	0.698	0.067	0.656	7.37	0.682	0.102	0.643

H, Heterozygosity; PIC, Polymorphism information content.

The cultigens and wild genotypes were also analysed separately to have a comparative view. In case of wild accessions (75), a total of 216 alleles were identified with an average of 6.17 alleles per locus, while in cultigens (203), 222 alleles were identified with an average of 6.34 alleles per locus. The number of alleles per locus in wilds ranged from 3 (PLC_105) to 10 (LC_03 and PBA_LC_1451) whereas it ranged from 3 (PBA_LC_1423, PBA_LC_829) to 12 (LC_04) in cultigens. The gene diversity and PIC values in wilds varied between 0.404–0.851 and 0.342–0.835, with an average of 0.698 and 0.656 respectively. The primer which showed highest gene diversity and PIC values in wilds was PBA_LC_1451 while the lowest gene diversity and PIC values was observed for PBA_LC_1387 (Table 2). The gene diversity and PIC values in cultigens varied between 0.029–0.854 and 0.029–0.837, with an average of 0.619 and 0.578, respectively. The primer which showed highest gene diversity and PIC values was PBA_LC_1288. The lowest gene diversity and PIC values was observed for the primer PBA_LC_1423 (Table 2). The major allele frequency in wilds varied between 0.207 (PBA_LC_1366) to 0.733 (PBA_LC_1387) with a mean value of 0.419 (Fig 3). A representative profile of 48 wild genotypes (out of 75) with SSR marker PBA_LC_1403 is represented in S1 Fig. The major allele frequency of cultigens varied between 0.216 (PBA_LC_1288) to 0.985 (PBA_LC_1423) with a mean value of 0.416 (Fig 3). A representative profile of 48 cultivars (out of 203) with SSR marker PBA_LC_1480 is represented in S2 Fig.

### Cluster analysis using molecular markers and morpho-physiological traits

**Fig 4:**
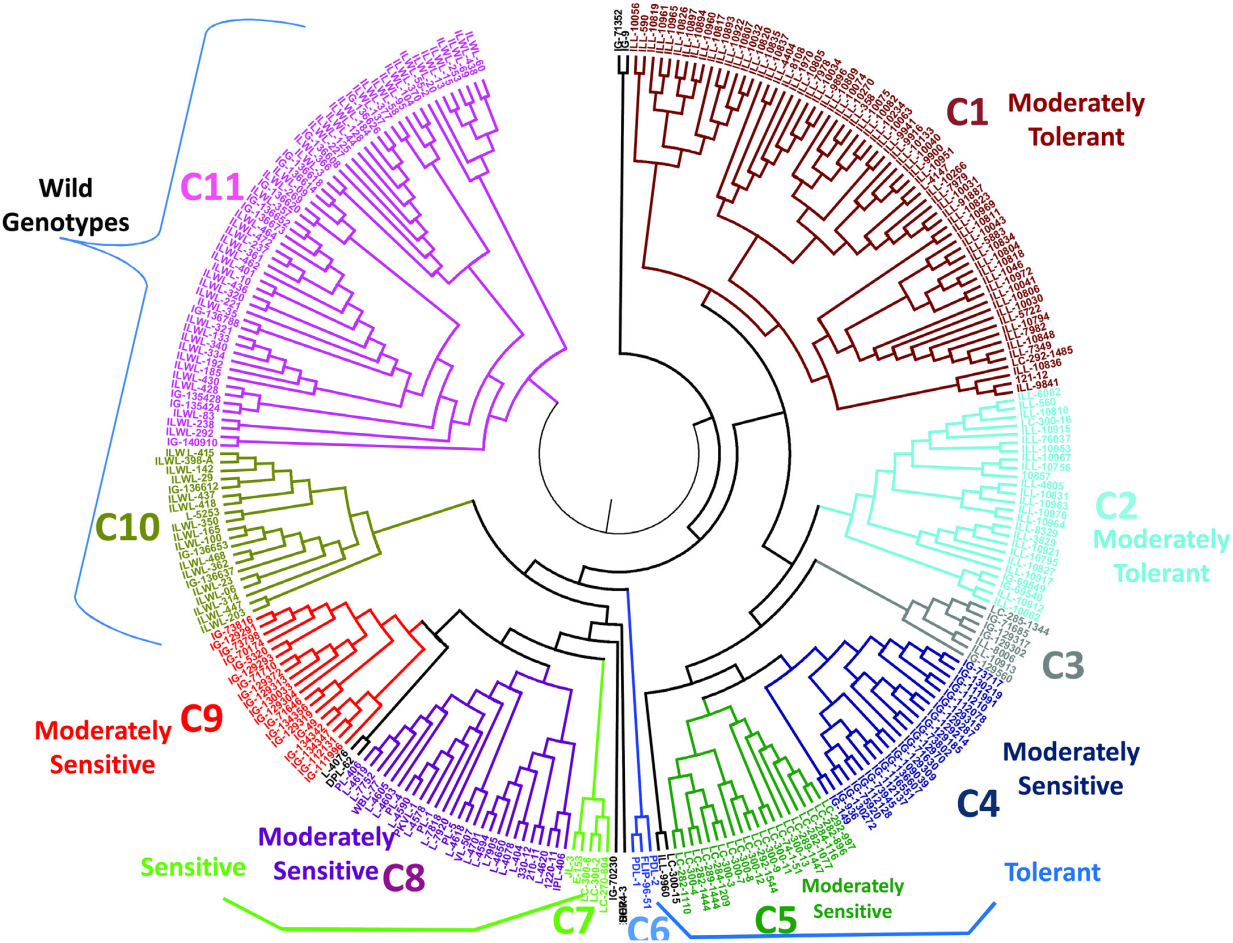
UPGMA tree based on dissimilarity index of 35 SSR markers for 278 lentil genotypes.

**Fig 5:**
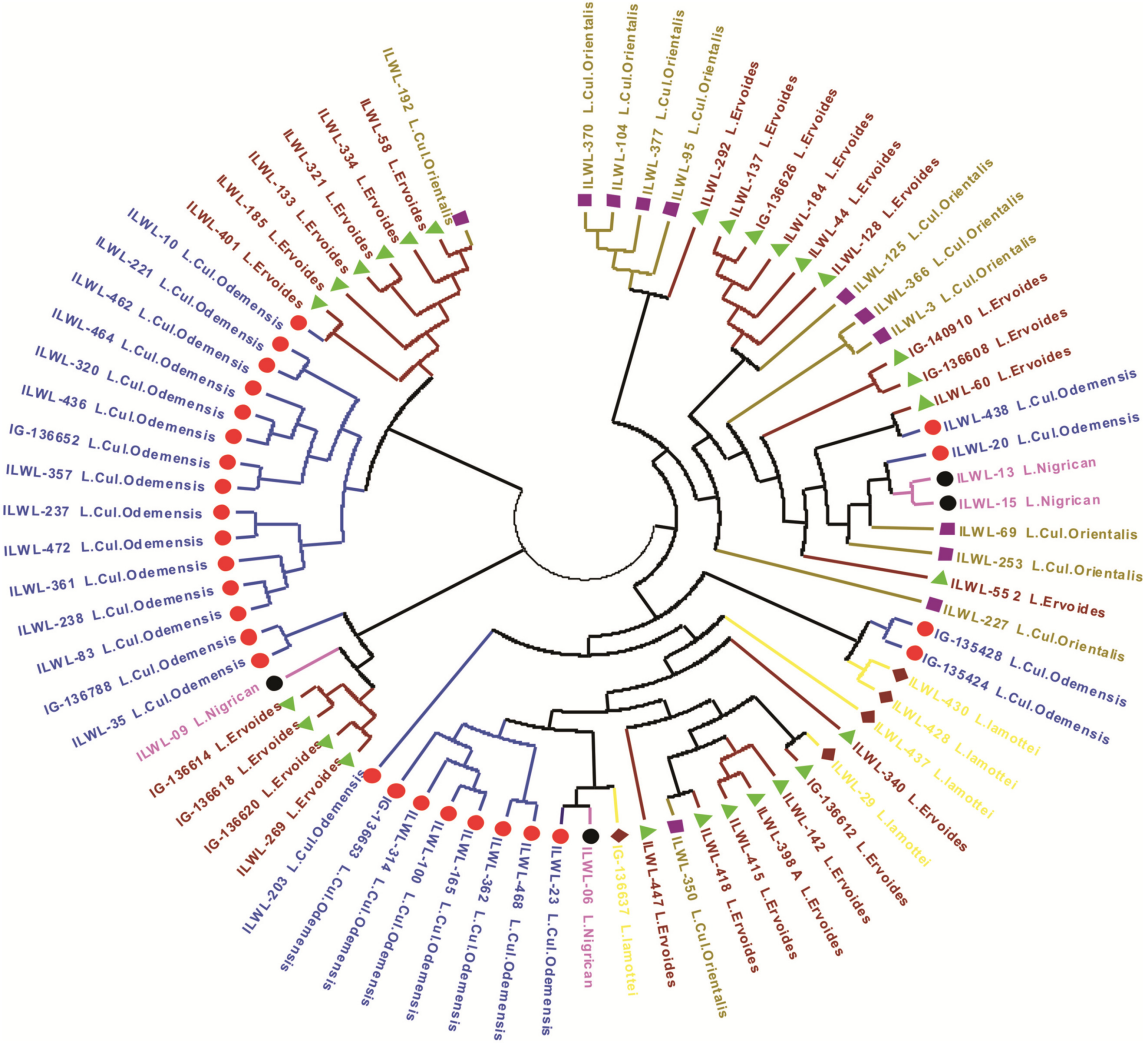
UPGMA tree based on dissimilarity index of 35 SSR markers for 75 wild genotypes.

The genetic relationships among lentil genotypes are presented in SSR based UPGMA tree (Fig 4 and S3 Fig). All the genotypes are grouped into eleven clusters (S3 Table). The genotypes IG-71352, IG-9, Sehore74-3, IG-70230, DPL-62, L-4076, LC-300-15 and ILL-9960 were distinct and not included into these clusters. Clusters 1 and 2 grouped moderately tolerant genotypes whereas clusters 4, 5, 8 and 9 formed groups with moderately sensitive genotypes. The genetic distance of the clusters ranged from 0.57 to 0.69 with an average of 0.60. Cluster 11 showed highest genetic distance (0.69) followed by cluster 6 (0.64); cluster 2 and 10 (0.61); cluster 1 (0.60); cluster 3 (0.59); cluster 5 and 4 (0.58); cluster 8 and 9 (0.57) (Table 3). Cluster analysis of only wild accessions was also done separately and their genetic relationships are presented in SSR based UPGMA tree (Fig 5). All the wild genotypes formed five major groups which were further sub-grouped according to species and subspecies *viz*. *L*. *culinaris orientalis*, *L*. *culinaris odemensis*, *L*. *nigricans*, *L*. *lamottei* and *L*. *ervoides*.

**Table 3:** Cluster means of seedling survivability, drought score (DS), reduction per cent of relative water content (RWC), chlorophyll content (Chl.), seed yield and genetic distance (GD) under drought stress conditions among the clusters of SSR markers.

	Genotypes	Survival%	DS	RWC	Chl.	Seed Yield	GD
**Group 1**	65	59.5^d^	1.8^b^	33.6^b^	47.1^b^	42.0^b^	0.60^a^
**Group 2**	25	57.6^d^	1.9^bc^	35.9^bc^	55.1^b^	43.9^b^	0.61^a^
**Group 3**	7	44.0^bc^	2.5^de^	60.7^d^	70.9^cd^	56.5^d^	0.59^a^
**Group 4**	23	38.8^b^	2.9^e^	73.2^e^	79.6^de^	70.7^e^	0.58^a^
**Group 5**	19	37.4^bc^	2.7^e^	74.3^e^	80.3^de^	69.9^e^	0.58^a^
**Group 6**	3	100.0^e^	0.1^a^	7.8^a^	15.0^a^	14.3^a^	0.64^b^
**Group 7**	5	23.3^a^	3.2^f^	80.5^e^	86.0^e^	75.3^e^	0.59^a^
**Group 8**	27	39.7^bc^	2.8^e^	76.1^e^	81.0^e^	71.6^e^	0.57^a^
**Group 9**	19	38.4^b^	2.8^de^	74.2^e^	79.0^de^	71.0^e^	0.57^a^
**Group 10**	20	49.8^bc^	2.4^cde^	56.2^d^	66.3^c^	59.5^d^	0.61^a^
**Group 11**	57	50.2^cd^	2.2^bcd^	44.2^c^	56.1^b^	49.9^c^	0.69^c^

Values within each column that do not share common letter are significantly different by Duncan’s post- hoc test at P≤0.05

**Fig 6:**
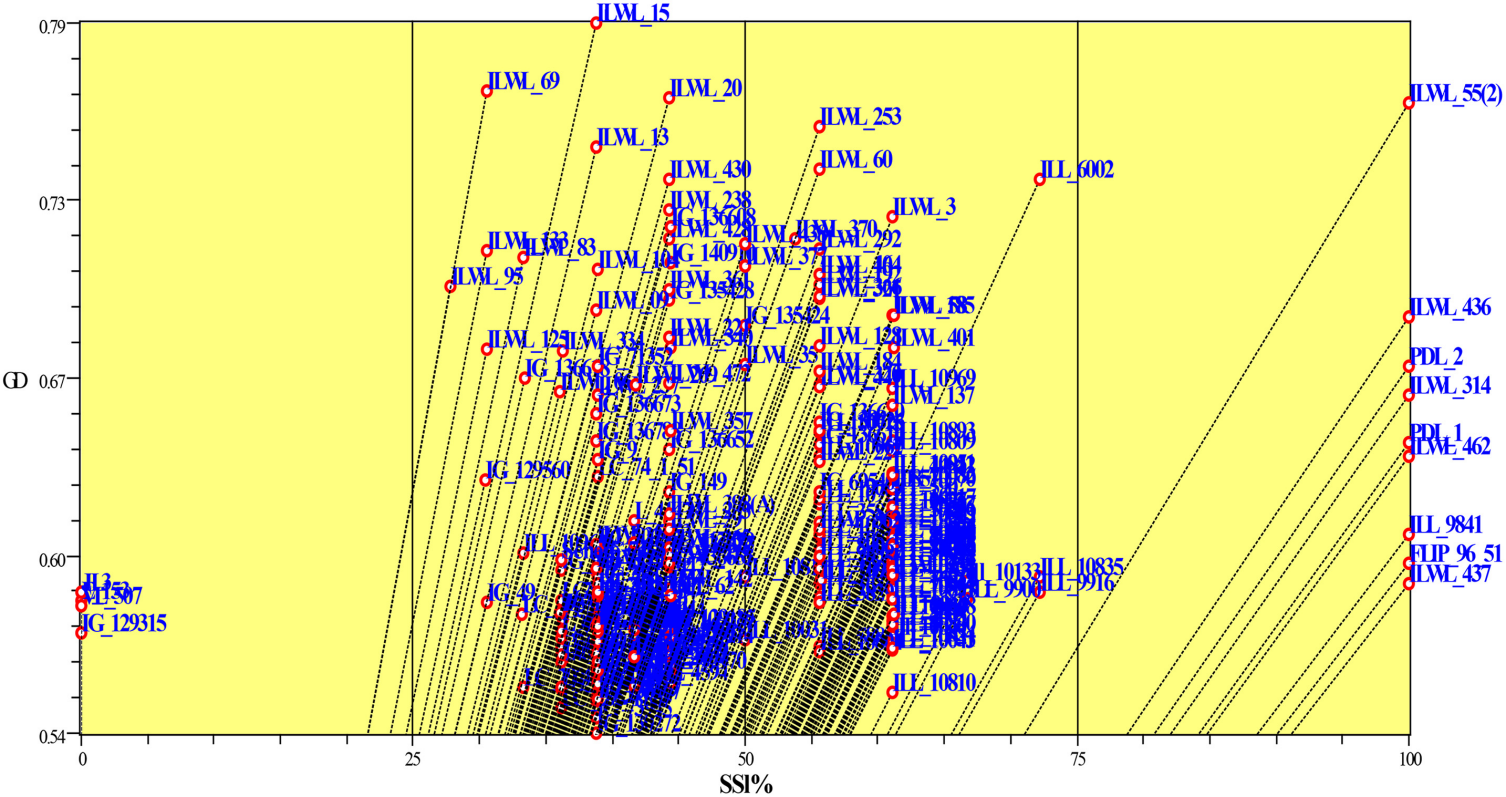
Correlation between genetic similarity index and taxonomic distance for seedling survivability.

The average reduction per cent of root length, shoot length, fresh and dry root and shoot weight, relative water content, chlorophyll content and seed yield along with values for seedling survivability (%), drought score and genetic distance under drought stress were calculated among the clusters categorized by SSR markers of all the genotypes. Among the SSR clusters there were wide range in values for most of the characters analyzed. Significant (*P* = 0.05) differences for the all characters were observed among the clusters. These parameters differed significantly for the genotypes of cluster 6 as compared to those of other clusters (Tables 3 and 4). The highest seedling survivability (100%) and lowest drought score (0.1), reduction in root length (21.4%), shoot length (43.5%), fresh and dry root (57.4%, 19.6%) and shoot weight (26.2%, 18.6%), relative water (7.5%) and chlorophyll contents (15.0%) were observed in the tolerant genotypes of cluster 6 compared to those of other clusters (Tables 3 and 4). These differences in the growth parameters and physiological traits may be due to strong drought tolerance among genotypes of cluster 6. The clusters based on SSR markers have been found to have relationship with the degree of drought tolerance. Most of the genotypes with the similar degree of drought tolerance were clustered into same groups. Correlation between genetic similarity index and taxonomic distance for seedling survivability was evaluated using Jaccard similarity index which clearly separated tolerant and sensitive genotypes (Fig 6).

**Table 4:** Cluster means of reduction per cent of root length (RL), shoot length (SL), fresh root weight (FRW), fresh shoot weight (FSW), dry root weight (DRW) and dry shoot weight (DSW) under drought stress conditions among the clusters of SSR markers

	Genotypes	RL	SL	FRW	FSW	DRW	DSW
**Group 1**	65	42.3^bc^	62.4^c^	71.6^cd^	47.5^bc^	38.9^b^	45.0^bcd^
**Group 2**	25	42.7^bc^	62.5^c^	72.0^cd^	48.4^c^	40.2^b^	44.7^bc^
**Group 3**	7	46.1^cd^	65.1^c^	74.9^de^	56.7^d^	53.5^c^	58.8^bcde^
**Group 4**	23	47.5^d^	66.6^c^	76.6^de^	63.7^e^	57.6^cd^	65.6^de^
**Group 5**	19	47.8^d^	67.6^c^	77.1^e^	65.2^e^	61.8^d^	68.1^e^
**Group 6**	3	21.4^a^	43.5^a^	57.4^a^	26.2^a^	19.6^a^	18.6^a^
**Group 7**	5	53.7^e^	72.2^d^	81.4^f^	64.2^e^	58.5^cd^	69.3^e^
**Group 8**	27	47.5^d^	66.5^c^	76.2^de^	64.4^e^	61.5^d^	70.8^e^
**Group 9**	19	47.5^d^	66.9^c^	76.1^de^	63.0^e^	59.6^d^	64.6^cde^
**Group 10**	20	39.7^b^	61.5^b^	69.8^bc^	42.0^b^	41.5^b^	49.1^bcd^
**Group 11**	57	38.6^b^	59.6^b^	68.0^b^	43.7^bc^	39.4^b^	44.1^b^

Values within each column that do not share common letter are significantly different by Duncan’s post- hoc test at P≤0.05

### Population Structure analysis

**Fig 7:**
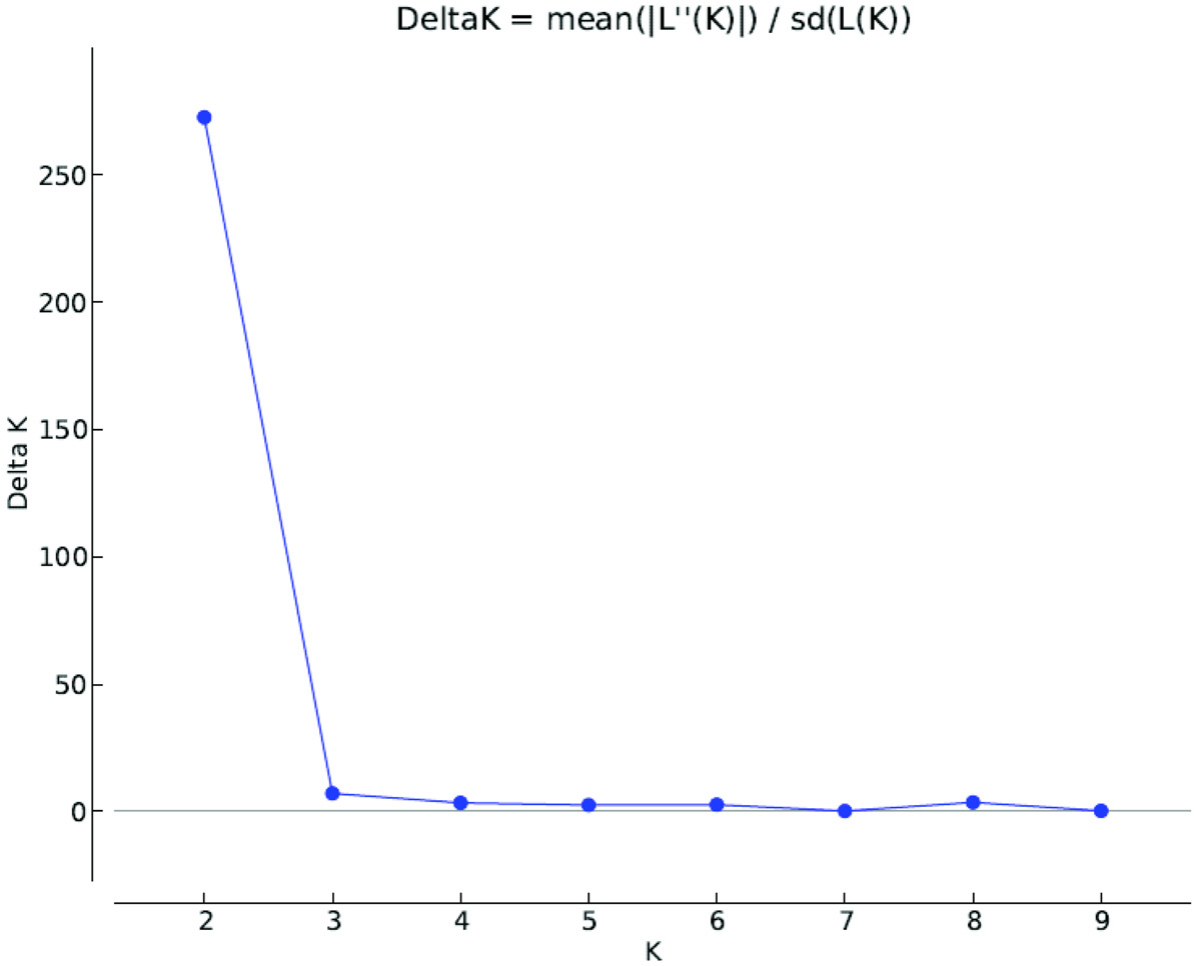
Evanno plot describing estimation of cultigens and wild genotypes ofgenus *Lens* using LnP(D) derived Δ k for k from 1 to 10.

**Fig 8:**
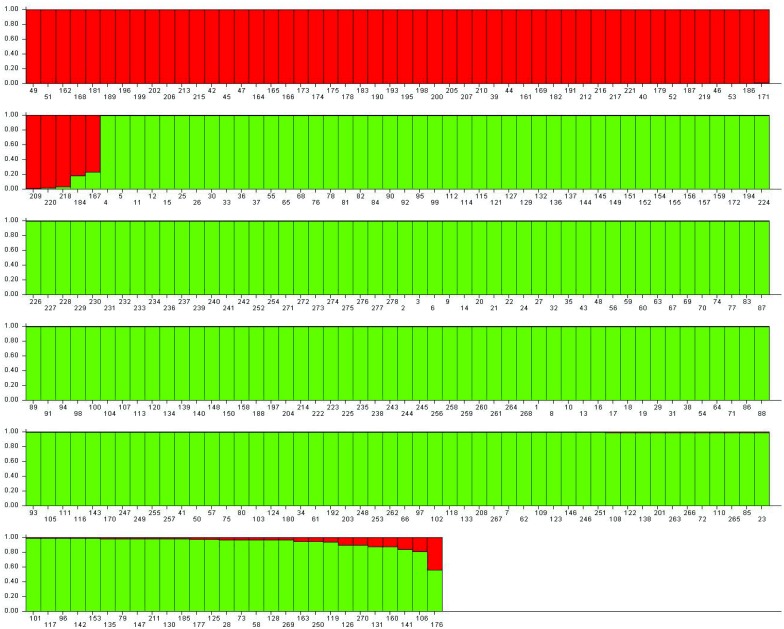
Model based population structure plot with K = 2, using structure with 35 SSR markers. Colour codes: Population I red (Wild accessions) and population II green (Cultivars).

The population structure of the 278 lentil genotypes was estimated using STRUCTURE v2.3.3 software based on 35 SSR markers. The optimum K value was determined by using Structure Harvester, where the highest peak was observed at delta K = 2 (Fig 7). The number of subpopulations (K) was identified based on maximum likelihood and delta K (dK) values, with accessions falling into two subgroups (Fig 8). Using a membership probability threshold of 0.80, 55 genotypes were assigned to subgroup (SG) 1, two hundred twenty three genotypes to SG 2 and 45 genotypes were retained in the admixed group (AD). The relationship between subgroups derived from STRUCTURE explained that SG 1 comprised of wild types and SG 2 consisted of cultivars mainly. This indicated that the population structure was in accordance with clustering of lentil genotypes formed using UPGMA tree.

### Principle Co-ordinate analysis

**Fig 9:**
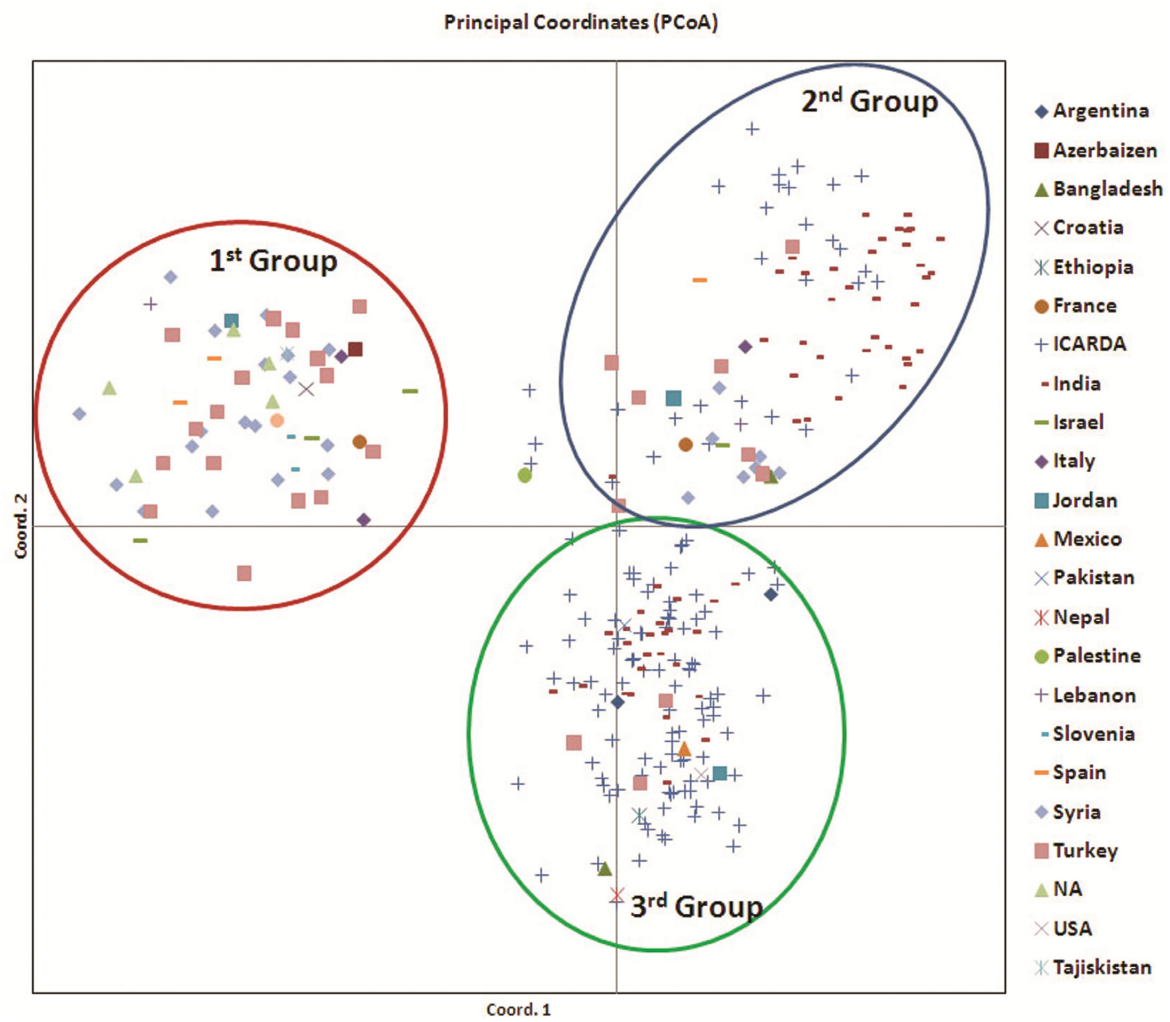
Principal co-ordinate analysis of all genotypes originating from 22 countries based on SSR data.

Principle Co-ordinate analysis (PCA) based on origin formed three major population groups. Group 1^st^ included accessions from Europe and Middle East including countries Israel, Turkey, Spain, Syria, Slovenia, Italy, Croatia, Tajikistan, Lebanon, Jordan. Group 2^nd^ included accessions mainly from ICARDA, India and Turkey. Third group consisted of accessions from ICARDA, India, Turkey, Argentina, Bangladesh, Nepal, Ethiopia, USA and Mexico. Further the accessions from ICARDA and India were dispersed in both 2^nd^ and 3^rd^ groups (Fig 9).

### Evaluation of most contrasting genotypes in clusters 6 and 7 under drought stress in field conditions

#### Rain-fed conditions

**Fig 10:**
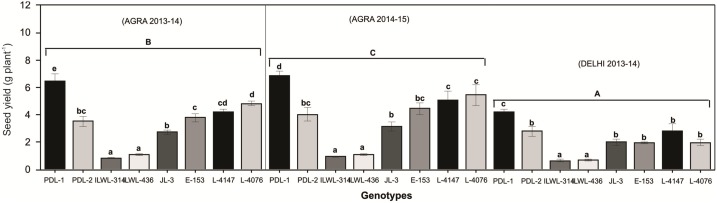
Seed yield of lentil genotypes grown under rain-fed condition at Agra and Delhi during 2013–14 and 2014–15. Data shown are mean ± SEm. Vertical bars that do not share common small letters are significantly different within year/location while different capital letters indicates significant differences across locations/years by Duncan’s post hoc test at P≤0.05.

Preliminary screening performed on the whole collection at National Phytotron Facility, Indian Agricultural Research Institute, New Delhi, revealed a broad range of response to drought stress among the tested *Lens* material (Fig 1), which allowed the selection of contrasting genotypes to be further validated under rain-fed conditions during 2013–14 and 2014–15 at Central Soil and Water Conservation Research Institute, Agra, India and 2013–14 at Indian Agricultural Research Institute, New Delhi, India. A wide degree of variation was obtained across the locations. The tolerant cultivars (PDL-1 and PDL-2) and wild (ILWL-314 and ILWL-436) accessions showed 10.5 to 26.5% and 7.5% to 15.6% reduction in seed yield per plant under rain-fed conditions, respectively. On the other hand, sensitive genotypes ‘JL-3’ and ‘E-153’ recorded the maximum seed yield reduction *i*.*e*. 50.6 to 65.5% (Fig 10).

#### Severe moisture stress condition

**Fig 11:**
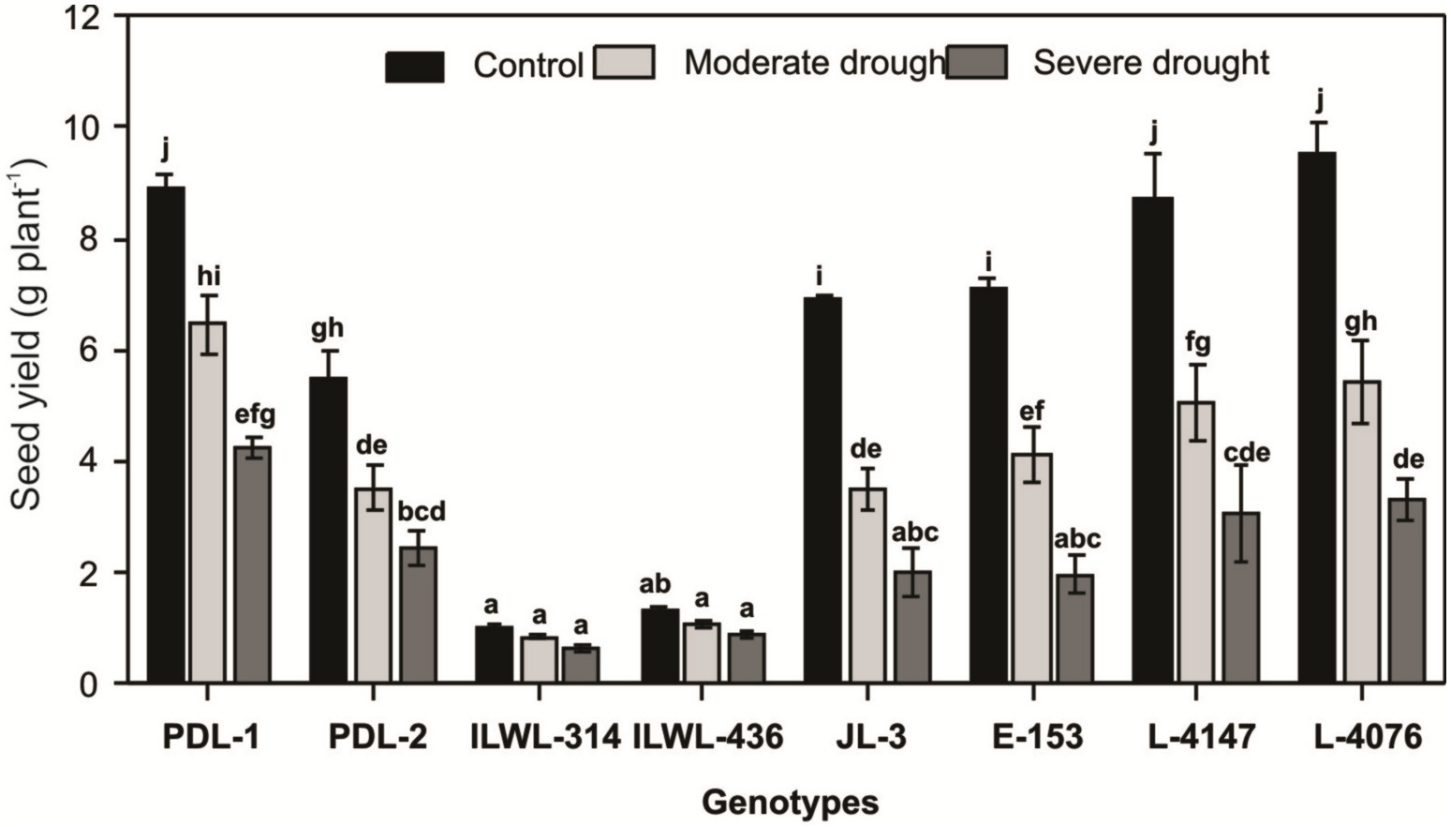
Seed yield of lentil genotypes grown under control, moderate and severe drought conditions. Data shown are mean ± SEm. Vertical bars that do not share common letters are significantly different by Duncan’s post hoc test at P≤0.05.

Six cultivated genotypes (PDL-1, PDL-2, L-4076, L-4147, JL-3 and E-153) and two wild accessions (ILWL-314, ILWL-436) were again evaluated under severe moisture stress condition using polythene tunnels which allowed the crop to grow solely on stored soil moisture at Indian Agricultural Research Institute, New Delhi during 2013–14. Drastic reduction for seed yield levels was observed in all the genotypes in relation to control. The lowest reduction of seed yield was there in tolerant cultivated (‘PDL-1 and PDL-2’) and tolerant wild accessions (ILWL-314 and ILWL-436). The reductions in seed yield in response to stress treatment were 48–49% in ‘PDL-1, and ‘PDL-2’, 30.5–45.3% in ‘ILWL-314’ and ‘ILWL-436’ and 66–70.0% in ‘JL-3’ and ‘E-153’. The reduction in moderately tolerant genotypes was intermediate (Fig 11).

## Discussion

Drought stress adversely affects every aspect of plant growth and metabolism in lentil which increases with increasing duration of water deficit conditions. For improvement of drought tolerance in lentil, knowledge about contrasting genotypes is essential which can be achieved through molecular assortment and characterization of genotypes for drought response. Due to breeding and domestication of lentil in different parts of the world, there has been a considerable increment in its gene pool apart from the restored genetic constitution of wild species. The genotypes (cultivars, breeding lines, landraces, germplasm collection and wild types) used in the present study were collected from different regions of the world (Table 1) so that a wide genome potential can be explored. A close genetic relationship between parental genotypes is a common problem in drought tolerance breeding programs that restricts the success of selection in segregating populations, especially when the underlying physiological characters are targeted. The genotypic assortment in the present study have grouped the similar genotypes, which may allow lentil breeders to select most contrasting lines for developing genetic linkage map based on drought stress and for further introgression of resistant gene(s) in high yielding cultivars. Several genetic diversity analyses has been conducted among cultivated and wild lentils using various molecular marker systems like RFLP, AFLP and RAPD markers, but SSRs were least explored among them [49]. Further, many of the morphological and molecular markers were used in previous studies to characterize the genome in many crop plants like bread wheat, rice, maize, cassava etc. for drought tolerance [50–53], but few studies has been undertaken in case of lentil so far (30, 31).

For efficient selection of genotypes for any abiotic stress, precise phenotyping is an equally important aspect. Screening for drought tolerance can be done under laboratory, green house and field conditions; however, hydroponic assay among them is the most easy, simple and economical method. Hydroponic method allows screening in controlled environment and a large number of lines per plants in a small area can be effectively screened. Another advantage of this method is that it is non-destructive; thereby selected plants can be transferred to pots or fields for further assessment of drought tolerance at subsequent stages of growth [16]. Using this method, all the plants which were exposed to air for 5 h showed severe wilting except for tolerant wild accessions (ILWL-314 and ILWL-436) which were comparatively less affected. Significant differences were observed when all the plants were re-immersed in the nutrient solution for 12 hours. Only tolerant genotypes were recovered, whereas sensitive ones did not show any recovery. This depicted that drought stress resulted in complete breakdown of plant metabolism in the sensitive genotypes whereas tolerant ones showed resurrection. Drought recovery was measured by important parameters *i*.*e*. drought score and seedling survivability which provided an instant description about drought reactions of genotypes [16].

In present study we observed reduction in fresh and dry root and shoot weights under drought stress conditions. Similar root and shoot traits variability related to drought tolerance in lentil was also observed by Idrissi *et al*. where they found that drought stress significantly reduced root and shoot characteristics compared to well-watered conditions in RILs derived from a cross between ILL 6002 and ILL 5888 [33]. Ashraf and Iram also found that imposition of water deficit conditions on *Phaseolus vulgaris* and *Sesbania aculeata* plants had significant inhibitory effect on fresh and dry weights of both root and shoot as well as on shoot length [54]. Similarly, Hu *et al*. observed that shoot fresh weight of maize plants was reduced as compared to control under drought stress [55]. Further, decline in shoot and root lengths in response to drought may be due to decreased cell elongation rate caused due to effect of water shortage on growth promoting hormones which in turn, led to a decrease in cell turgor, cell volume and eventually cell growth [56]. This could also be due to restriction of water and nutrients transport through xylem and phloem vessels [57]. The decline in fresh and dry weights of roots and shoots can be due to influence of water on regulation of photosynthetic enzymes and growth promoting hormones, which regulates dry matter production [58]. The rapid recovery of the tolerant genotypes following re-watering also suggests that there was no loss of reaction centres instead they may have played a regulatory role in recovery after drought stress.

The physiological response to drought stress was measured based on chlorophyll and relative water content. Relative water content decreased significantly in all the genotypes in response to water stress but its reduction was significantly lower in tolerant genotypes both under non stress and water stress conditions. It is suggested that due to high relative water content physiochemical and biochemical processes are performed efficiently under water stress conditions in tolerant genotypes than the sensitive ones. Higher relative water content has been reported to be associated with higher photosynthetic pigments, membrane stability index, osmolytes and antioxidant activities [59]. Open stomata causes more transpiration and subsequently the relative water content of plants reduce. Under this condition the genotypes loses a lot of water and particularly if drought is prolonged for a long duration plant recovery is impossible and plant death will occur. Tolerant genotypes maintain water in their leaves by stomatal closure and consequently reduction in transpiration rate [60]. Lentil genotypes at the IARI location had higher reduction of seed yield per plant than those grown at the CSWRTI, Agra. The lower reduction of seed yield may be because of favourable moisture as well as longer life cycle of plants at CSWCRTI, Agra. From both the locations, ‘PDL-1’ and ‘PDL-2’ (tolerant cultivated), ‘ILWL-436’ and ‘ILWL-314’ (tolerant wild) had lowest reduction of seed yield per plant than those from ‘JL-3’ and ‘E-153’ (sensitive genotypes) under rain-fed conditions (Fig 10). Further, under severe moisture conditions at IARI, during 2013–14, there was drastic reduction in seed yield levels in all the genotypes, though tolerant genotypes *viz*. ‘PDL-1’ and ‘PDL-2’ among cultigens and ‘ILWL-314’ and ‘ILWL-436’ among wilds recorded lowest reduction in the field (Fig 9). Under hydroponic condition also, tolerant cultivated and wild genotypes showed less reduction in seed yield per plant than the sensitive ones. Moreover, in tolerant wild genotypes ‘ILWL-314’ and ‘ILWL-436’ there was minimum reduction in seed yield per plant under both hydroponic and field conditions, *i*.*e*. rainfed and severe drought. Utilization of these tolerant cultivated and wild genotypes in breeding programme can be helpful for development of drought tolerant genotypes.

Based on the cluster analysis, out of 15 tolerant cultivars, three *viz*. ‘PDL-1’, ‘PDL-2’ and ‘FLIP-96-51’ were grouped in cluster 6 while sensitive ones were mainly grouped into cluster 7. Growth parameters like reduction in root length, shoot length, fresh root weight, fresh shoot weight, dry root weight, dry shoot weight and physiological parameters like relative water content and chlorophyll content were also low in the genotypes of cluster 6, as compared with those of other clusters. On the other hand, reduction in these traits in genotypes of cluster 7 was comparatively high. The clustering of drought contrasting genotypes into clusters 6 and 7 may be useful to produce better segregants for drought tolerance. Similar distinction of drought tolerant and sensitive genotypes into different clusters have also been reported in many other crops like wheat, barley, rice etc. [61–67].

Allelic diversity analysis in this study revealed that an average of 7.37 alleles per locus were amplified in 278 lentil genotypes and their PIC value ranged from 0.299–0.836 which was higher when compared to the previous reports of SSR markers in lentil [26, 64]. Results of gene diversity using 35 markers indicated that wild accessions (mean gene diversity: 0.698) had higher gene diversity than the cultivars (mean gene diversity: 0.619). This indicated that wild gene pool may have potential unique genes. The genetic diversity within wild separated itself from the cultivars which were supported from the results of both the cluster analysis using PowerMarker software and population structure developed using STRUCTURE software. Similarly, when Dikshit *et al*. studied the genetic diversity and population structure among 86 accessions of three *Lens* species using EST and genomic SSRs, they found that the genetic diversity was greater in wild species as compared to the cultivated *L*. *culinaris* subsp. culinaris genotypes [65]. In the present study, the combined dendrogram of wilds and cultivars, separated wilds in 10^th^ and 11^th^ group and none of the wild accession was found in any other group which solely comprised of cultivars only. Population structure did separate most of the wild accessions within one sub group but some of the wild types were also found to be dispersed in another sub group comprising of cultivars mainly (92%). But, no cultivar was found in the sub group of wilds. This indicated that genetic constituent of wild varied from that of cultivars which could be attributed to fact that cultivars have undergone extensive inbreeding and domestication over the decades while, in wild species their original genetic constitution has been maintained. Cluster analysis of wild accessions had further grouped them on the basis of species and sub-species which indicated that genetic relatedness within wild accessions is conserved within a particular species and sub-species.

Wild species are fully exposed to stressful edaphic and climatic conditions and therefore are reservoir of useful genes which can be utilized for improvement of cultivated genotypes. Some of the wild accessions belonging to sub species *odemensis* were found to be tolerant to drought stress. Cross-ability of these genotypes with cultivars can be exploited for introgression of drought associated gene(s) in high yielding cultivars which will help in widening the genetic base of cultivated genotypes. Although, there is difficulty in obtaining hybrids from crosses between cultivars with wilds, the crossability between *L*. *culinaris orientalis* and *L*. *odomensis* with cultivated genotypes have already been established before by Maehlbour *et al*. and Fratini and Ruiz [18, 19]. Ovule and embryo rescue have also been used as alternative techniques to overcome interspecific incompatibility in lentil [66, 67]. In this study, most of the wild accessions belonging to *L*. *ervoides* were found to be moderately tolerant to drought stress while some of the genotypes like ‘ILWL 55(2)’ were found to be tolerant which can be used for obtaining interspecific crosses following the above mentioned techniques. Also attempts could be made to develop direct interspecific crosses between cultivated and selected drought stress tolerant wild (*L*. *ervoides*) accessions and viability of seeds could be checked as there are reports where some of the interspecific crosses between cultivars and selected *L*. *ervoides* genotypes have produced viable progeny. Tullu *et al*. have successfully produced an interspecific recombinant inbred line (RIL) population designated LR-26 from a cross of *L*. *culinaris* ‘Eston’ and *L*. *ervoides* (Brign.) Grande accession IG 72815 where they examined the inheritance of resistance to *Colletotrichum truncatum* (Schwein.) and studied the genetic variation in agronomic traits and their relationships to each other [68].

In present study, it was found that the cultivars were grouped according to drought reactions. The tolerant and sensitive genotypes were mainly grouped in 6^th^ and 7^th^ cluster respectively. The other groups comprised of either moderately tolerant or moderately sensitive genotypes. Wild accessions showed both moderately tolerant and moderately sensitive reactions within their clusters. Assortment of most of the cultivars according to drought reaction can be explained by the fact that specific phenotype and their corresponding genotypes are focussed during the inbreeding and domestication process. This accumulates the gene of interest and eliminates the less relevant ones. The fact that wild accessions which are grouped according to its species and sub-species rather than drought reaction also indicates the same as there is no domestication and inbreeding involved in case of wild types. Further, fluctuations in the population sizes and genetic bottlenecks effects have caused genetic drift of cultivated and wild species which may have further added to overall genetic distinction in wild and cultivated lentil populations. Similarly, distinction of wild types from cultivars has previously been reported in lentil, pigeonpea, and pearl millet [69–71]. Also, significant differences among clusters were observed in respect of morpho-physiological and reproductive traits (Tables 3 and 4).

Lentil breeding programs to develop drought tolerant lines (‘PDL-1’ and PDL-2) from the materials introduced from ICARDA is ongoing at Indian Agricultural Research Institute, New Delhi, India. These two lines were bred under the same habitat; therefore their genetic background is identical. Also they were found identical based on SSR markers, grouped into same cluster in dendrogram (Fig 4) and were ranked on top for drought tolerance under field conditions (Fig 11). In one of the recent study where mapping population was developed from cross between JL-3 x PDL-1 (two genotypes falling in cluster 7 and 6 respectively in this study), seven SSR were found to be associated with drought tolerance and were linked together (Singh *et al*., unpublished), which further proves that drought tolerance responses are genetically controlled.

The important acquisition in this study could be applied to lentil breeding programs for improving drought tolerance using SSR markers. Improvement in drought tolerance can be achieved by selecting parental genotypes based on SSR markers. Drought tolerance of lentil genotypes/cultivars could be improved by inter-crossing genotypes of cluster 6 and 7 as there are perceptible differences for morpho-physiological parameters among them. The crossing between the genotypes from clusters 6 and 7 would help in evolution of better segregants for conserving drought tolerance in lentil.

The summary in this study provided some implications for engineering drought tolerance in lentil using SSR clusters.

1. Improving drought tolerance can be achieved by selecting parental genotypes before inter-crossing based on SSR markers. With the aid of SSR makers, different drought tolerance components can be combined by inter-crossing the genotypes from different clusters. For example genotypes in Cluster 6 have better RWC and chlorophyll contents and seedling survivability than those in Cluster 7. Crosses between these two clusters would yield segregants with improved drought tolerance.
2. The diverse drought tolerance mechanisms among SSR clusters indicated that these mechanisms are genetically controlled. Therefore, identifying different drought tolerance components and pyramiding them into drought tolerant cultivars is suggested. Also, the success in pyramiding different drought tolerance components into a cultivar can be increased by using SSR markers as identification of these tolerance components using conventional methods is strenuous.
3. Wild species have reservoir of useful genes but they are seldom crossable with cultivated types. To overcome this barrier, various methods like embryo rescue have previously been used, although these are not very much practical approaches from breeder’s view point as they require some sophisticated conditions. Propitiously, wilds belonging to *odemensis* and *orientalis* species are crossable with cultivated genotypes. Moreover, some of these were found to be tolerant to drought stress. Crossability of these genotypes with cultivars can be used for introgression of drought associated gene(s) in high yielding cultivars.

## Supporting Information

S1 Fig Electrophoretic profile of PCR amplified products of microsatellite marker PBA_LC_1403 in 48 wild genotypes. Base pairs (bp), 100bp DNA Ladder (L).
(TIF)

S2 Fig Electrophoretic profile of PCR amplified products of microsatellite marker PBA_LC_1480 in 48 cultivars. Base pairs (bp), Marker (M = 100bp DNA Ladder).
(TIF)

S3 Fig UPGMA tree based on dissimilarity index of 35 SSR markers for 278 lentil genotypes along with bootstrap values. (TIF)

S1 Table Maximum and minimum values of temperature, relative humidity and total rainfall in the growing season 2013–14 of the studied genotypes at IARI, New Delhi. (DOCX)

S2 Table Maximum and minimum values of temperature, relative humidity and total rainfall in the growing season 2013–14 and 2014–15 of the studied genotypes at CSWCRTI, Agra. (DOCX)

S3 Table Genotypes in various clusters based on simple sequence repeat markers. (DOCX)
