# Supplementary figures and images for: Molecular Assortment of Lens Species with Different Adaptations to Drought Conditions Using SSR Markers
Source: PLoS One. 2016 Jan 25;11(1):e0147213. doi: 10.1371/journal.pone.0147213 (PMC4726755; doi:10.1371/journal.pone.0147213)

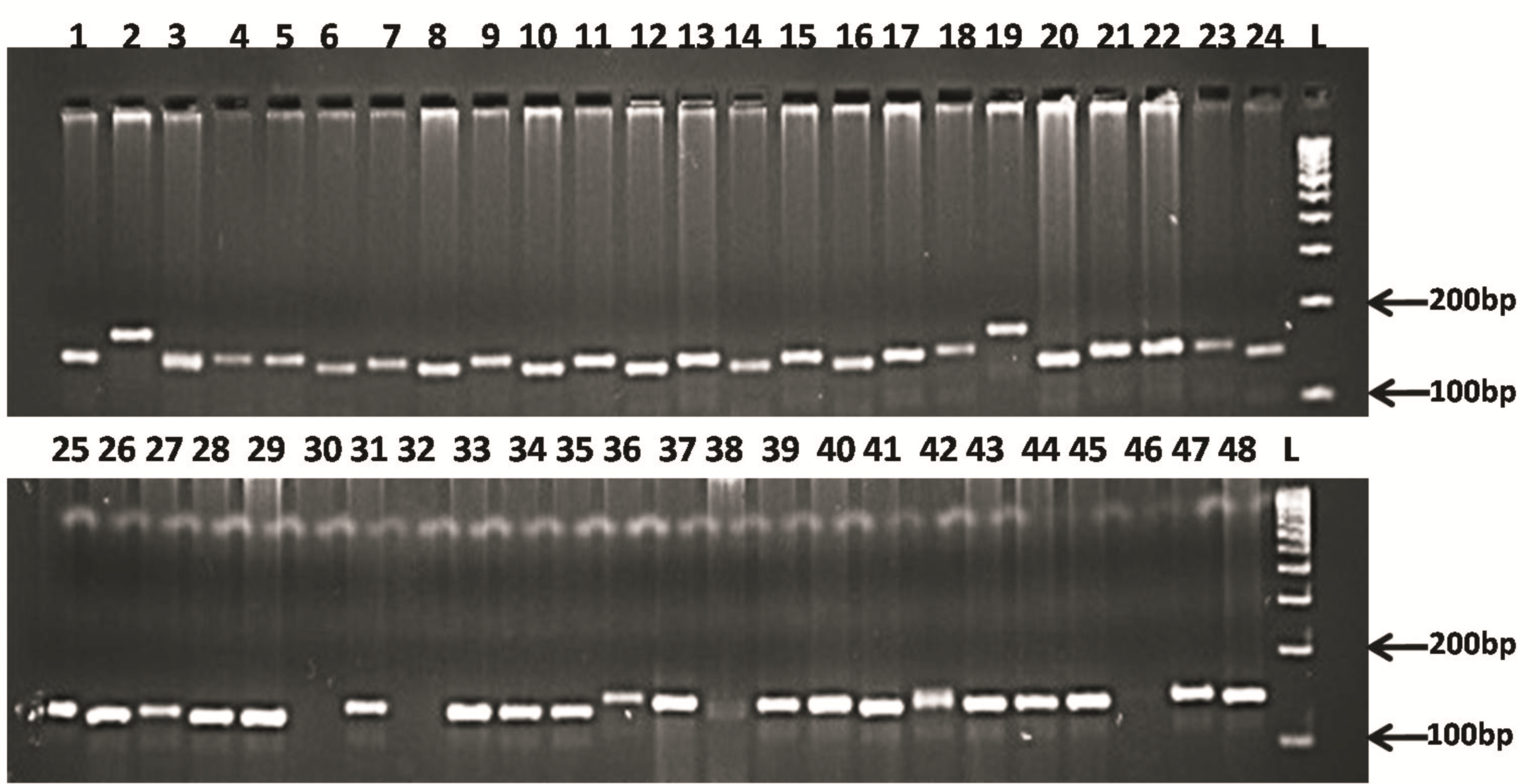

Supplement: S1 Fig — Base pairs (bp), 100bp DNA Ladder (L). (TIF) [file pone.0147213.s001.tif]

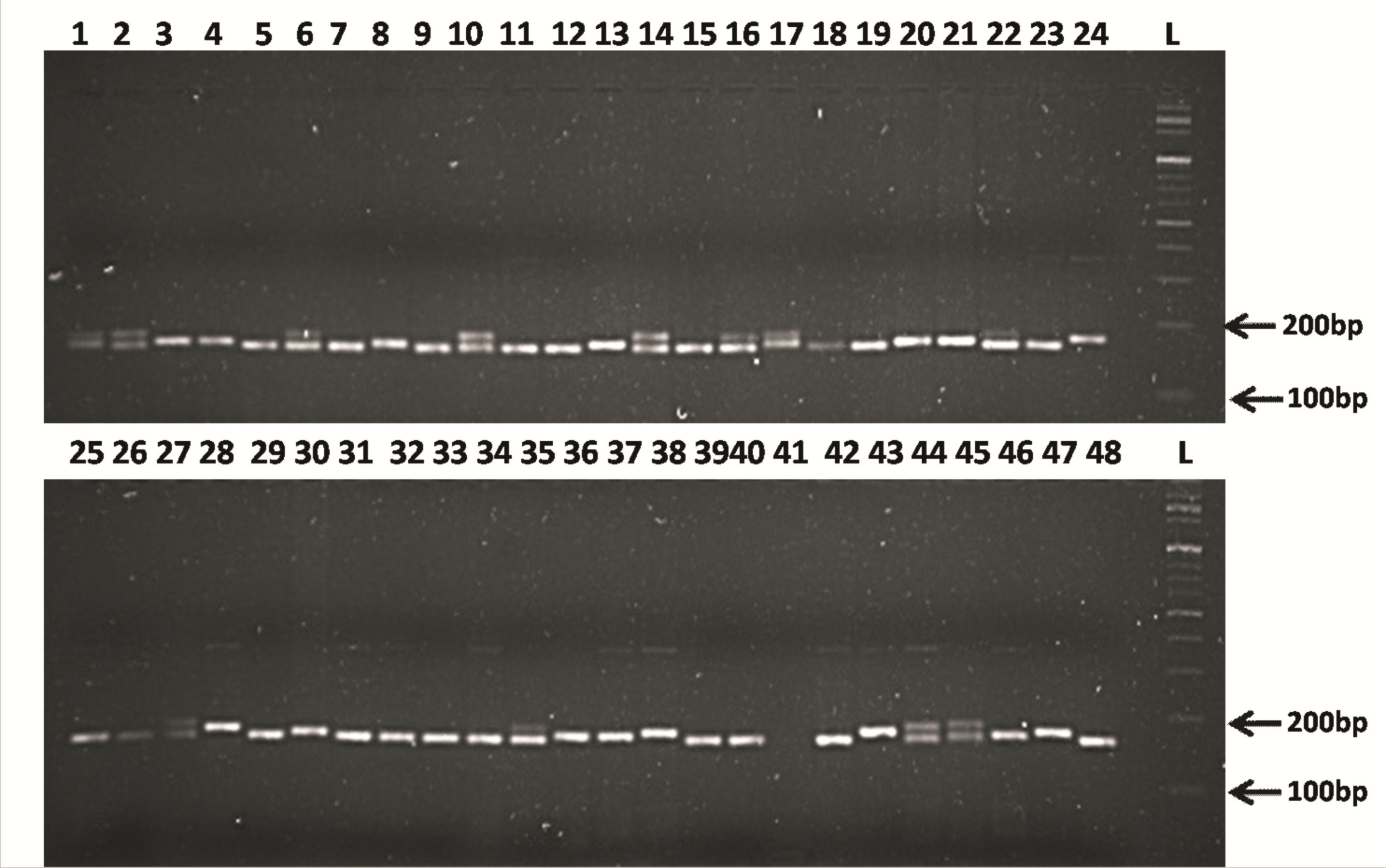

Supplement: S2 Fig — Base pairs (bp), Marker (M = 100bp DNA Ladder). (TIF) [file pone.0147213.s002.tif]

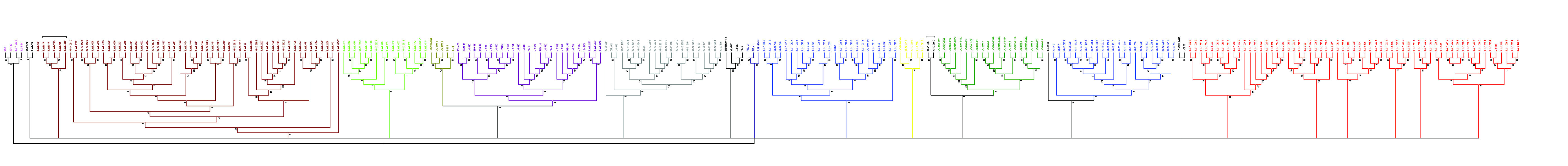

Supplement: S3 Fig — (TIF) [file pone.0147213.s003.tif]
