# Supplementary material for: Molecular Assortment of Lens Species with Different Adaptations to Drought Conditions Using SSR Markers
Source: PLoS One. 2016 Jan 25;11(1):e0147213. doi: 10.1371/journal.pone.0147213 (PMC4726755; doi:10.1371/journal.pone.0147213)
Supplement: S1 Table — (DOCX) [file pone.0147213.s004.docx]

| Month | Temperature (^o^C) | | Relative Humidity (%) | | Rainfall (mm) |
| --- | --- | --- | --- | --- | --- |
|  | Maximum | Minimum | Maximum | Minimum |  |
| November | 26.9 | 9.9 | 90.9 | 48.2 | 0.0 |
| December | 22.4 | 7.1 | 93.8 | 55.9 | 0.2 |
| January | 18.6 | 6.8 | 96.6 | 66.5 | 0.6 |
| February | 21.4 | 7.5 | 96.0 | 63.1 | 2.3 |
| March | 27.2 | 12.7 | 90.0 | 48.1 | 2.0 |
| April | 34.8 | 17.9 | 73.4 | 40.1 | 0.5 |
| Average | 25.2 | 10.3 | 90.1 | 53.7 | 1.1 |
