# Supplementary material for: Molecular Assortment of Lens Species with Different Adaptations to Drought Conditions Using SSR Markers
Source: PLoS One. 2016 Jan 25;11(1):e0147213. doi: 10.1371/journal.pone.0147213 (PMC4726755; doi:10.1371/journal.pone.0147213)
Supplement: S2 Table — (DOCX) [file pone.0147213.s005.docx]

| Season 2013-14 | | | | | |
| --- | --- | --- | --- | --- | --- |
| Month | Temperature (^o^C) | | Relative Humidity (%) | | Rainfall (mm) |
|  | Maximum | Minimum | Maximum | Minimum |  |
| November | 28.2 | 10.3 | 91 | 33 | 0 |
| December | 28 | 10 | 92 | 33 | 0 |
| January | 17.7 | 7.7 | 95 | 76 | 61.4 |
| February | 22.6 | 8.9 | 95 | 50 | 45.4 |
| March | 29.2 | 13.6 | 87 | 37 | 22 |
| April | 37.2 | 18.4 | 64 | 22 | 6.1 |
| Average | 27.2 | 11.5 | 87.3 | 41.8 | 22.5 |
| Season 2014-15 | | | | | |
| November | 30.1 | 11.6 | 82 | 27 | 0 |
| December | 21.8 | 7 | 87 | 54 | 11.5 |
| January | 16.8 | 7.2 | 94 | 72 | 37.4 |
| February | 26.3 | 11.5 | 86 | 45 | 0 |
| March | 28.9 | 14.3 | 91 | 45 | 85.2 |
| April | 35.6 | 20 | 71 | 32 | 43 |
| Average | 26.6 | 11.9 | 85.2 | 45.8 | 29.5 |
