# Supplementary material for: Molecular Assortment of Lens Species with Different Adaptations to Drought Conditions Using SSR Markers
Source: PLoS One. 2016 Jan 25;11(1):e0147213. doi: 10.1371/journal.pone.0147213 (PMC4726755; doi:10.1371/journal.pone.0147213)
Supplement: S3 Table — (DOCX) [file pone.0147213.s006.docx]

| **Group** | **No.** | **Genotype** |
| --- | --- | --- |
| **Cluster 1** | 65 | ILL-10056, ILL-590, ILL-10819, ILL-10961, ILL-10965, ILL-10826, ILL-10897, ILL-10894, ILL-10960, ILL-10817, ILL-10893, ILL-10922, ILL-10807, ILL-10032, ILL-10820, ILL10835, ILL-10837, ILL-4404, ILL-8108, ILL-1970, ILL-10805, ILL-7978, ILL-10034, ILL-9896, ILL-10809, ILL-10074, ILL-10270, ILL-358, ILL-10075, ILL-10082, ILL10234, ILL-10063, ILL-9941, ILL-9916, ILL-10133, Ill-10040, ILL-9900, ILL-10951, L-4147, ILL-10266, ILL-7979, ILL-10031, ILL-91887, ILL-10823, ILL-10969, ILL-10811, ILL-10043, ILL-5883, ILL-10834, ILL-10804, ILL-10818, ILL-1046, ILL-10972, ILL-10041, ILL-10806, ILL-10030, ILL-5722, ILL-10794, ILL-7982, ILL-10848, ILL-7349, LC-292-1485, ILL10836, 121/12, ILL-9841 |
| **Cluster 2** | 25 | ILL-6002, ILL-560, ILL-10810, LC-300-16, ILL-10915, ILL-76037, ILL-10953, ILL-10967, ILL-10756, 10857, ILL-4605, ILL-10831, ILL-10963, ILL-10970, ILL-10964, ILL-8329, ILL-3829, ILL-10921, ILL-10795, ILL-10827, ILL-10917, IG-69549, IG-69540, ILL-10812, ILL-10062 |
| **Cluster 3** | 7 | LC-285-1344, IG-71685, IG-129317, IG-129302, ILL-8006, ILL-10913, IG-129560 |
| **Cluster 4** | 23 | IG-73717, IG-130219 , IG-111991, IG-11210, IG-112078 , IG-129315, IG-129287, IG-129214, IG-129185, IG-73802, IG-12970, IG71630, IG-129309, IG-109039, IG-136607, IG-116551, IG-112137, IG-73945, IG-112128, IG-75920, IG-130272, IG-936, IG-149 |
| **Cluster 5** | 19 | LC-292-997, LC-282-896, LC-284-116, LC-282-1077, LC-289-1447, LC-300-13, LC-74-1-51, LC-300-11, LC-300-9, LC-292-1544, LC-300-12 , LC-300-8, LC-300-7 , LC-300-3, LC-284-1209, LC-289-1444, LC-282-1444, LC-300-4, LC-282-1110 |
| **Cluster 6** | 3 | PDL-2, FLIP-96-51, PDL-1 |
| **Cluster 7** | 5 | LC-270-804, LC-300-2, LC-300-6, E-153, JL3 |
| **Cluster 8** | 27 | IPL-406, 1220-11, L-4620, 210-11, 330-12, L-404, L-4078, L-4650, L-7905, L-4594, L-4701, VL-507, L-4618, PL-5, L-7920, L-7818, PL-1, L-4578, PKVL-1, L-4590, PL-4, L-4603, ILL-4605, WBL-77, L-7752, L-4619, PL-406 |
| **Cluster 9** | 19 | IG-111996, IG-112131, IG-134347, IG-134342, IG-49, IG-129319, IG-134356, IG-71646, IG-129304, IG-130033, IG-129313, IG-29372, IG-71710, IG-129293, IG-5320, IG-70174, IG-73798, IG-129291, IG-73816 |
| **Cluster 10** | 20 | ILWL-203, ILWL-447, ILWL-314, ILWL-06, ILWL-23, IG-136637, ILWL-362, ILWL-468, IG-136653, ILWL-100, ILWL-165, ILWL-350, L-5253, ILWL-418, ILWL-437, IG-136612, ILWL-29, ILWL-142, ILWL-398(A), ILWL-415 |
| **Cluster 11** | 57 | IG-140910, ILWL-292, ILWL-238, ILWL-83, IG-135424, IG-135428, ILWL-428, ILWL-430, ILWL-185, ILWL-192, ILWL-334, ILWL-340, ILWL-133, ILWL-321, IG-136788, ILWL-35, ILWL-221, ILWL-320, ILWL-436, ILWL-10, ILWL-401, ILWL-462, ILWL-361, ILWL-237, ILWL-472, ILWL-464, IG-136673, IG-136652, ILWL-357, IG-136620, ILWL-269, ILWL-09, IG-136614, IG-136618, ILWL-3, ILWL-366, IG-136608, ILWL-227, ILWL-125, ILWL-44, ILWL-128, ILWL-184, IG-136626, ILWL-137, ILWL-377, ILWL-58,ILWL-95, ILWL-104 , ILWL-370, ILWL-55(2), ILWL-20, ILWL-13, ILWL-15, ILWL-253, ILWL-69, ILWL-438, ILWL-60 |
